# Antagonistic properties against *Fusarium sporotrichioides* and glycosylation of HT-2 and T-2 toxins by selected *Trichoderma* strains

**DOI:** 10.1038/s41598-024-55920-x

**Published:** 2024-03-11

**Authors:** Marta Modrzewska, Dominik Popowski, Lidia Błaszczyk, Łukasz Stępień, Monika Urbaniak, Marcin Bryła, Benedikt Cramer, Hans-Ulrich Humpf, Magdalena Twarużek

**Affiliations:** 1 Department of Food Safety and Chemical Analysis, Prof. Waclaw Dabrowski Institute of Agricultural and Food Biotechnology-State Research Institute, Rakowiecka 36, 02-532 Warsaw, Poland; 2 Plant Microbiomics Team, Institute of Plant Genetics, Polish Academy of Sciences, 60-479 Poznan, Poland; 3 Plant-Pathogen Interaction Team, Institute of Plant Genetics, Polish Academy of Sciences, 60-479 Poznan, Poland; 4 Institute of Food Chemistry, University of Münster, Corrensstr. 45, 48149 Münster, Germany; 5 Department of Physiology and Toxicology, Faculty of Natural Sciences, Institute of Experimental Biology, Kazimierz Wielki University, Chodkiewicza 30, 85–064 Bydgoszcz, Poland

**Keywords:** Sustainable agriculture, Food safety, Metabolomic profiling, Fungal interactions, Toxin glycosylation, Metabolomics, Fungi, Pathogens

## Abstract

The present study assessed the ability of *Trichoderma* to combat *F. sporotrichioides*, focusing on their antagonistic properties. Tests showed that *Trichoderma* effectively inhibited *F. sporotrichioides* mycelial growth, particularly with *T. atroviride* strains. In co-cultures on rice grains, *Trichoderma* almost completely reduced the biosynthesis of T-2 and HT-2 toxins by *Fusarium*. T-2 toxin-α-glucoside (T-2-3α-G), HT-2 toxin-α-glucoside (HT-2-3α-G), and HT-2 toxin-β-glucoside (HT-2-3β-G) were observed in the common culture medium, while these substances were not present in the control medium. The study also revealed unique metabolites and varying metabolomic profiles in joint cultures of *Trichoderma* and *Fusarium*, suggesting complex interactions. This research offers insights into the processes of biocontrol by *Trichoderma*, highlighting its potential as a sustainable solution for managing cereal plant pathogens and ensuring food safety.

## Introduction

Cereals are an essential raw material in food and feed production^1^. The changing climate, particularly in recent years, is posing a huge challenge to food production worldwide. These problems are not only related to the severity of weather events during the growing season of cereal plants and may also affect the increased occurrence of plant pathogen infections during flowering^2^. In this context, protecting cereal plants from pathogenic fungi and insects (often vectors in the spread of fungal diseases) takes on particular importance^3^. The current global trend assumes reducing the use of chemical pesticides towards sustainable agriculture development and food security assurance. Thus, new solutions in crop protection are required. One proposed strategy is using living organisms as biocontrol agents against pathogens^4^. These biocontrol agents function through a mechanism of spatial competition with the pathogens, thereby inhibiting the growth of these harmful organisms^5^. Hitherto, a diverse array of organisms, including bacteria and fungi, have demonstrated efficacy in this realm of plant disease management^6^.

In recent years, significant attention has been directed towards the capabilities of the *Trichoderma* fungus to effectively combat species of the *Fusarium* genus^7^*. Trichoderma* species are known for their comprehensive roles both in biostimulation, through enhancing plant growth and resilience to environmental stressors, and in antagonistic interactions via mycoparasitism, where they feed plant pathogens, as well as antibiosis associated with the secretion of biochemical compounds to inhibit or exterminate competing fungal species^8^. These fungi are potential sources of secondary metabolites exhibiting a wide array of antimicrobial activities, encompassing both antibacterial and antifungal properties, and biosynthesis of these substances depends on the species, strain, and environmental conditions^9^. According to Tian et al^10^., *Trichoderma* spp. are not only responsible for their ability to compete with pathogens through the biosynthesis of metabolites but can also participate in the biotransformation of mycotoxins produced by pathogenic fungi, especially those of *Fusarium* genus.

*Fusarium sporotichoides* is a pathogen of cereal plants (including oats, barley, wheat) widely distributed over different temperature zones^11,12^. Toxigenic isolates of *F. sporotrichioides* are capable of biosynthesizing type A trichothecenes (T-2 toxin, HT-2 toxin, diacetoxyscirpenol, and neosolaniol), the toxic effects of which have been demonstrated in both animals and humans. Among others, the intake hazard is associated with the inhibition of protein synthesis, neurotoxicity, and immunotoxicity^13,14^. Mycelial growth and mycotoxin biosynthesis by *F. sporotrichioides* depend mainly on temperature and water activity. The optimal temperature for *F. sporotrichioides* mycelial growth ranges between 25 and 30 °C and the optimal water activity ranges from 0.980 to 0.995^11,15^. However, for the biosynthesis of mycotoxins, the optimal temperature is lower, ranging from 10 to 15°C^11^.

The study aimed to evaluate the antagonism of selected *Trichoderma* species and strains towards *F. sporotrichioides*. The metabolic profile changes were investigated to understand the underlying interactions between *Trichoderma* and the pathogen. The ability of *Trichoderma* strains to inhibit T-2 and HT-2 toxin biosynthesis and their potential for toxin biotransformation were assessed. To the best of our knowledge, this is the first report characterizing the potential of *Trichoderma* to biotransform HT-2 and T-2 toxins to glucoside derivatives.

## Results

**Figure 1:**
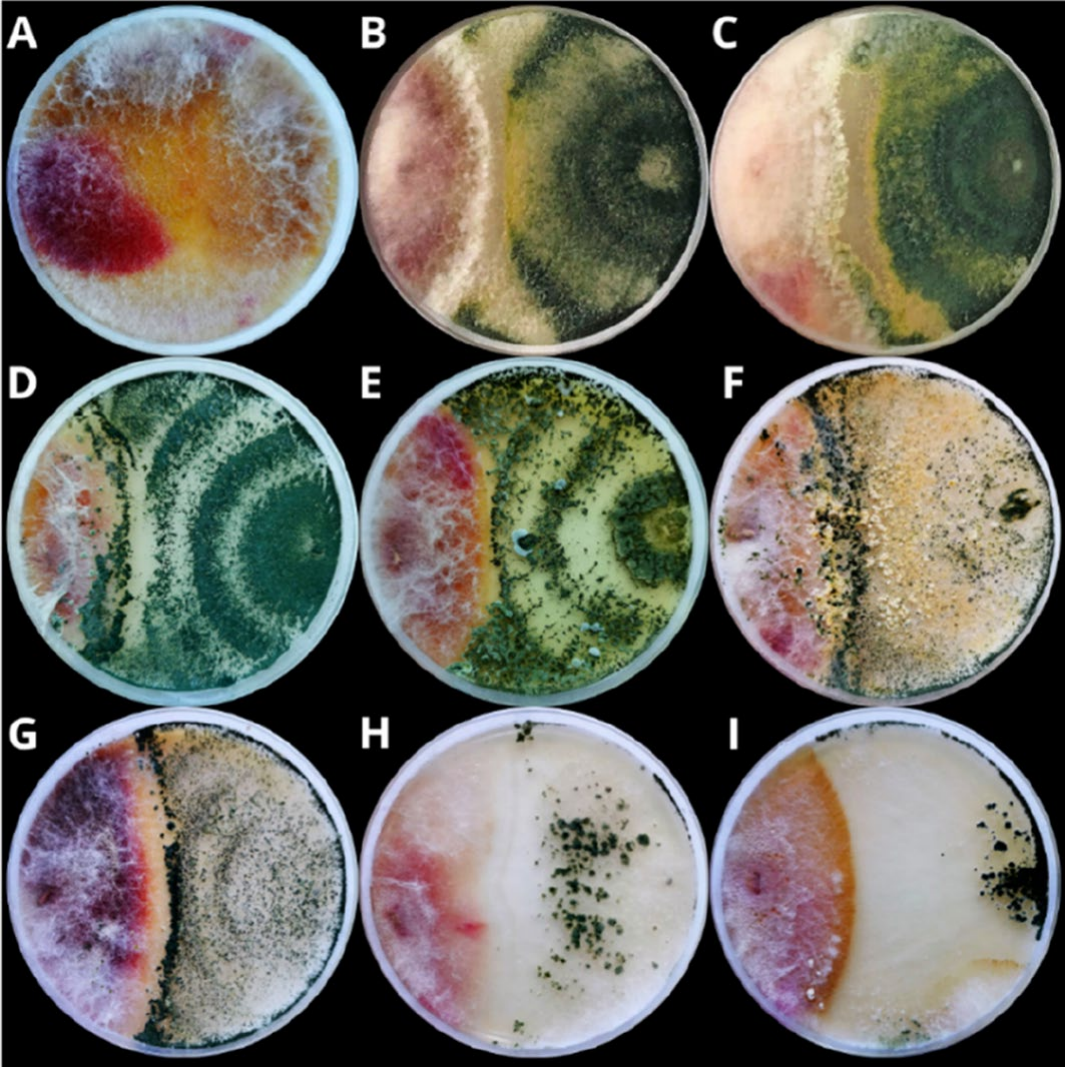
Fungal morphology of *F. sporotrichioides* 2006a in dual culture assay after 5 days incubation. (**A**) *F. sporotrichioides* 2006a grown alone (control), (**B**) *F. sporotrichioides* 2006a versus *T. atroviride* AN153, (**C**) *F. sporotrichioides* 2006a versus *T. atroviride* AN215; (**D**) *F. sporotrichioides* 2006a versus *T. atroviride* AN523, (**E**) *F. sporotrichioides* 2006a versus *T. atroviride* AN705, (**F**) *F. sporotrichioides* 2006a versus *T. viridescens* AN508; (**G**) *F. sporotrichioides* 2006a versus *T. viridescens* AN609, (**H**) *F. sporotrichioides* 2006a versus *T. viride* AN355, (**I**) *F. sporotrichioides* 2006a versus *T. viride* AN690.

**Figure 2:**
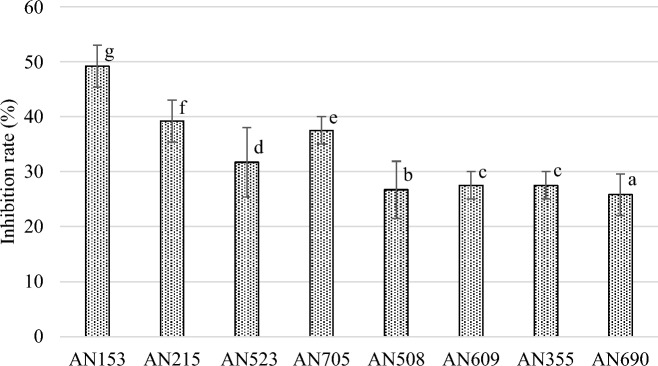
Estimated inhibition of mycelial growth of *F. sporotrichioides* by *Trichoderma* after five days of co-incubation on PDA medium. Bars with the same letter are not significantly different according to α = 0.01.

Co-culture tests on agar media showed that all *Trichoderma* strains significantly inhibited the growth of *F. sporotrichioides* 2006a (Figs. 1 and 2). Furthermore, the presence of *Trichoderma* greatly affected the morphological changes of the mycelium of *F. sporotrichioides* 2006a. After five days of co-incubation, an average degree of pathogen growth inhibition by the antagonist was assessed, ranging from 25 to 49%, depending on the *Trichoderma* species and strain.

Based on tests on solid rice substrate the ability of *Trichoderma* to inhibit the biosynthesis of selected mycotoxins by *F. sporotrichioides* 2006a was assessed. The concentration of HT-2 and T-2 toxins and their metabolites (T-2-3α-G and T-2-3β-G and HT-2-3α-G and HT-2-3β-G) in the culture media are shown in Table 1.

**Table 1:** Effect of different *Trichoderma* strains on the mycotoxin biosynthesis by *F. sporotrichioides* on solid substrates (rice kernels). T-2-3α-G: T-2 toxin-3α-glucoside, T-2-3β-G: T-2 toxin-3β-glucoside, HT-2-3α-G: HT-2 toxin-3α-glucoside, HT-2-3β-G: HT-2 toxin-3β-glucoside.

Trichoderma strain	Mycotoxin [mg/kg]					
	T-2	T-2-3α-G	T-2-3β-G	HT-2	HT-2-3α-G	HT-2-3β-G
Control	36.31^b^ ± 1.34	< LOD^a^	< LOD	23.21^b^ ± 1.57	< LOD^a^	< LOD^a^
AN153	0.02^a^ ± 0.01 (↓99%)	59.48^d^ ± 1.70	< LOD	< LOQ^a^ (↓100%)	5.70^c^ ± 0.14	< LOD^a^
AN215	0.05^a^ ± 0.01 (↓99%)	77.98^e^ ± 2.12	< LOD	< LOQ^a^ (↓100%)	6.41^d^ ± 0.13	1.81^b^ ± 0.01
AN523	< LOQ^a^ (↓100%)	0.46^a^ ± 0.01	< LOD	< LOQ^a^ (↓100%)	0.20^a^ ± 0.01	< LOD^a^
AN705	0.08^a^ ± 0.01 (↓99%)	4.17^bc^ ± 0.13	< LOD	0.16^a^ ± 0.03 (↓99%)	< LOQ^a^	< LOQ^a^
AN508	< LOQ^a^ (↓100%)	2.92^abc^ ± 0.07	< LOD	< LOQ^a^ (↓100%)	0.32^a^ ± 0.01	< LOD^a^
AN609	< LOQ^a^ (↓100%)	1.11^ab^ ± 0.13	< LOD	< LOQ^a^ (↓100%)	< LOD^a^	< LOD^a^
AN355	< LOQ^a^ (↓100%)	5.19^c^ ± 0.21	< LOD	< LOQ^a^ (↓100%)	0.58^b^ ± 0.04	< LOD^a^
AN690	< LOQ^a^ (↓100%)	2.55^abc^ ± 0.05	< LOD	< LOQ^a^ (↓100%)	0.28^ab^ ± 0.01	< LOD^a^

↓ decrease in mycotoxin concentration; for statistical analyses, 1/2 of the LOD (limit of detection)/LOQ (limit of quantification) values were used; values within columns followed by the same letter are not significantly different according to α = 0.01.

**Figure 3:**
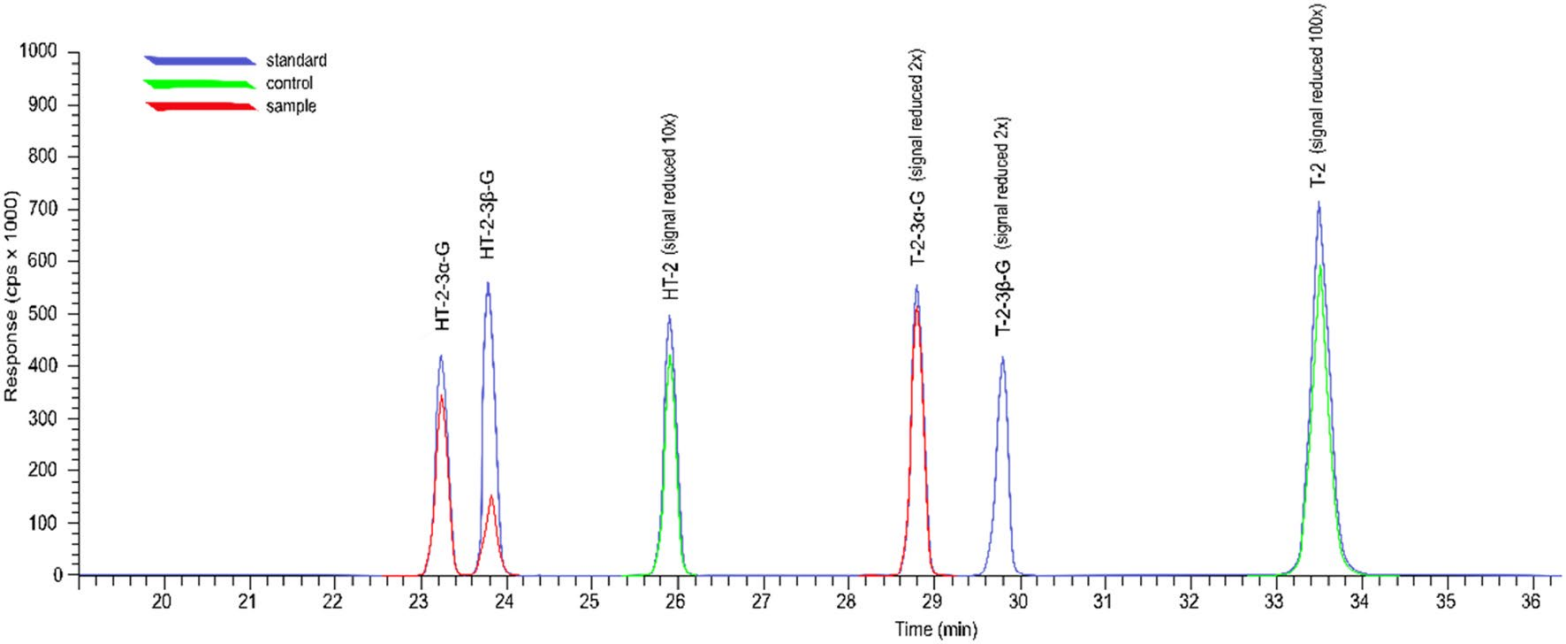
Estimated chromatogram including the response of the standard, the control sample (*F. sporotrichioides* 2006a grown alone), and the test sample (*F. sporotrichioides* 2006a /*T. atroviride* AN215).

In medium with the control strain *F. sporotrichioides* 2006a, T2 and HT-2 were identified at average levels of 36.31 and 23.21 mg/kg. For both toxins, the contents in the co-culture media were significantly lower than the control. These contents ranged from below the LOQ to 0.08 mg/kg for T-2 and from below the LOQ to 0.16 mg/kg for HT-2. In the co-culture medium, the presence of T-2-3α-G, HT-2-3α-G, and HT-2-3β-G were observed, while they were not in the control medium (Table 1; Fig. 3). The content of T-2-3α-G in these media ranged from 0.49 to 77.96 mg/kg and of HT-2-3α-G from below LOQ to 6.41 mg/kg, depending on the strain combination. HT-2-3β-G at an average level of 1.81 mg/kg was observed in the co-culture of *F. sporotrichioides* 2006a, and *T. atroviride* AN215; however, the presence of T-2-3β-G was not confirmed in any of the samples tested (> LOQ).

In order to determine the influence of *Trichoderma* strains on the growth of *F. sporotrichioides* in cultures on solid rice substrate, digital PCR (sPCR) analysis was performed. Based on the quantification of *F. sporotrichioides* DNA in individual cultures and in co-cultures with *Trichoderma*, the percentage of reduction in the pathogen's DNA concentration in the presence of the antagonist was estimated. The results of this analysis are presented in Table 2. It was documented that the presence of each of the tested *Trichoderma* strains had a significant impact (the number of DNA copies/µL for each sample was determined on the basis of the analysis of over 25,000 divisions—volumetric reactions) on the reduction of *F. sporotrichioides* DNA content, ranging from 84.8% (2006a/AN215) to 98.6% (2006a/AN355).

**Table 2:** *Fusarium sporotrichioides* DNA concentrations [copies/µL] in single cultures and co-cultures on solid rice substrate and the percentage [%] of reduction in patogen DNA content estimated based on digital PCR (dPCR) analysis.

Sample/NTC/Control	DNA concentration [copies/µL]	% of DNA concentration reduction
Variant1*		
DNAmix1	59.74	93.5
DNAmix2	9218	–
*Trichoderma*-mix	0.000	–
NTC_	0.000	–
Variant 2*		
2006a/AN609	0.634	91.7
2006a/AN508	0.387	94.9
2006a/AN355	0.110	98.6
2006a/AN690	0.614	91.7
2006a/AN705	0.389	94.9
2006a/AN215	1.164	84.8
2006a/AN153	0.327	95.7
2006a/AN523	0.479	93.7
2006a	7.644	–
*Trichoderma*-mix	0.000	–
NTC	0.000	–

*Variant 1: DNAmix1—DNA mix from all *Trichoderma* strains in co-cultures on rice with *Fusarium* samples; DNAmix2—DNA mix from all *Fusarium* in a single culture on rice samples; Trichoderma-mix—DNA mix from all *Trichoderma* in a single culture on rice samples used as a negative control; NTC—non-template control. Variant 2: a) DNA mix from 3 biological replicates for each *Trichoderma* strain in co-culture on rice with *Fusarium* samples; b) DNA mix from 3 biological replicates for *Fusarium* in a single culture on rice samples; c) DNA mix from all *Trichoderma* in single cultures on rice samples used as a negative control; d) NTC—non-template control.

**Figure 4:**
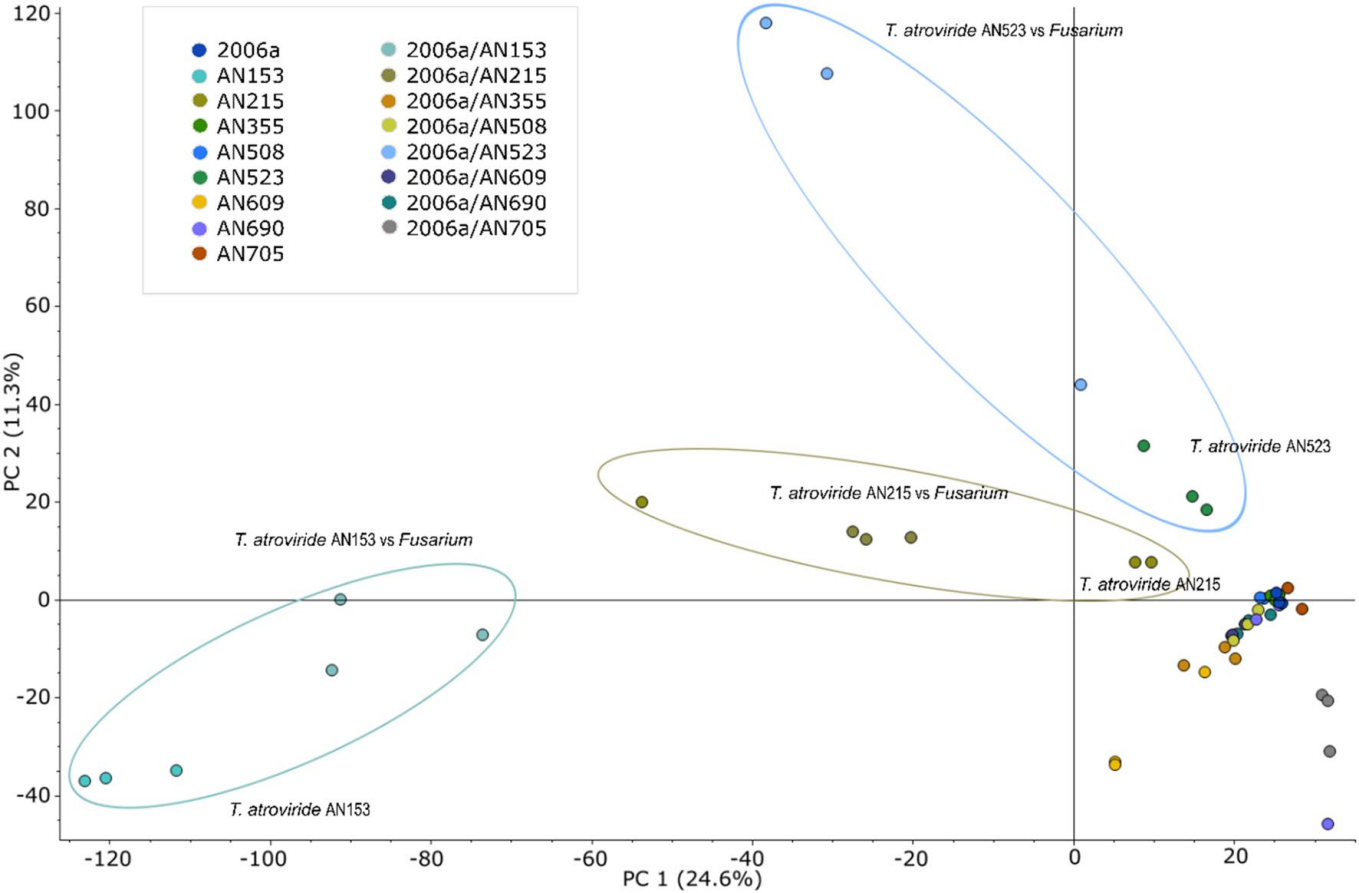
Principal component analysis showing clustering and separation of metabolite profiles in mono- and co-cultures; AN153: *T. atroviride* AN153, AN215: *T. atroviride* AN215, AN355; *T. viride* AN355, AN508: *T. viridescens* AN508, AN523: *T*. *atroviride* AN523, AN609*: T. viridescens* AN609, AN690: *T. viride* 690, AN705: *T*. *atroviride* AN705, 2006a: *F. sporotrichioides* 2006a control, 2006a/AN153: *F. sporotrichioides* 2006a with *T. atroviride* AN153; 2006a/AN215: *F. sporotrichioides* 2006a with *T. atroviride* AN215: 2006a/AN355; *F. sporotrichioides* 2006a with *T. viride* AN355: 2006a/AN508; *F. sporotrichioides* 2006a with *T. viridescens* AN508; 2006a/AN523: *F. sporotrichioides* 2006a with *T. atroviride* AN523; 2006a/AN609: *F. sporotrichioides* 2006a with *T. viridescens* AN609; 2006a/AN690: *F. sporotrichioides* 2006a with *T. viride* AN690; 2006a/AN705: *F. sporotrichioides* 2006a with *T. atroviride* AN705.

The metabolomic analysis allowed the extraction of nearly 50,000 molecular features (without filtering). Principal component analysis (PCA) identified groups of samples with significantly different metabolomic profiles compared to the control group—monocultures of *F. sporotrichioides* 2006a (Fig. 4). It was observed that the presence of an antagonist in the co-culture altered the qualitative and quantitative metabolomic profile of the pathogen, depending on the *Trichoderma* species and isolate. A list of 196 metabolites was created with molecular masses ranging from 153 to 1092 Da. They were present in the media in which both *F. sporotrichioides* 2006a were cultured with antagonists (TF group) and significantly differed from the control media (groups T and F). Namely, the mean area of individual compounds was significantly higher (TF/T and TF/F ratios higher than 5, simultaneously, *p* < 0.05) than in the control samples, or the presence of these metabolites in the media resulted from a co-culture incubation (they were absent in the control samples, Table 1S).

Subsequently, the filters were set to obtain a list of metabolites characteristic of either *Fusarium* or *Trichoderma* strains. A total of 276 metabolites present in the control cultures and the co-cultures of *F. sporotrichioides* 2006a and *Trichoderma* were extracted (Table S2). The criterion chosen to filter the data was a statistically significant difference between the mean peak areas of the analyzed substances (p < 0.05) and a ratio of these areas greater than 10 when considering differences between the two control samples (T vs. F or F vs. T). Probable metabolites specific to the *Trichoderma* strains used (160 metabolites, T/F ratio > 10, *p* < 0.05) as well as *F. sporotrichioides* 2006a (115 metabolites, F/T ratio > 10, *p* < 0.05) were identified similarly. Among the 160 metabolites attributed to *Trichoderma*, 49 were more abundant, and for 21 of them, the relative content in the co-cultures was lower than in the *Trichoderma* control samples. None of the metabolites considered specific to *F. sporotrichioides* 2006a increased its relative content in the co-culture samples relative to the control *Fusarium* monoculture. Almost all others (114 of 115) decreased their relative content under coincubation.

After setting the peak intensity threshold to 5.0 × 10^5^, the data was processed to find m/z values of possible T-2, HT-2, neosolaniol (NEO), and diacetoxyscirpenol (DAS) metabolites based on in silico biotransformations generated by the software. The list was filtered to obtain features assigned as characteristic of TF samples (TF/T and TF/F greater than 5, every *Trichoderma* strain isolate analyzed separately, Figure S1, Table S3). Eleven metabolites present in co-cultures obtained using strains AN508 and AN705 fragmented to ions characteristic to mycotoxins. Four metabolites were probably DAS derivatives, fragmenting to *m/z* 229.124, *m/z* 107.090 and/or *m/z* 81.070 (Figure S2- numbers 8–11). Two metabolites were tentatively identified based on fragmentary ions at *m/z* 271.095 and *m/*z 263.056 (Figure S2- numbers 4,5). However, the most unexpected result was the detection of 5 unknown metabolites that had fragments at *m/z* 249.113, *m/z* 231.102, *m/z* 203.107 and *m/z* 125.060 (calculated formulas C_14_H_17_O_4_; Δ 0.614 ppm, C_14_H_15_O_3_; Δ 0.739 ppm, C_13_H_15_O_2_; Δ 0.324 ppm, and C_7_H_9_O_2_; Δ 0.404 ppm, respectively), which are ions characteristic of B group trichothecenes, such as deoxynivalenol (DON) (Figure S2- numbers 1–3; 6,7). Furthermore, the metabolite with *m/z* 646.307 (Rt = 23.251), present in all co-cultures, was identified as a T2-3α-G fragmented to ions *m/z* 305.139 and 215.107 (Figure S2- number 12).

**Figure 5:**
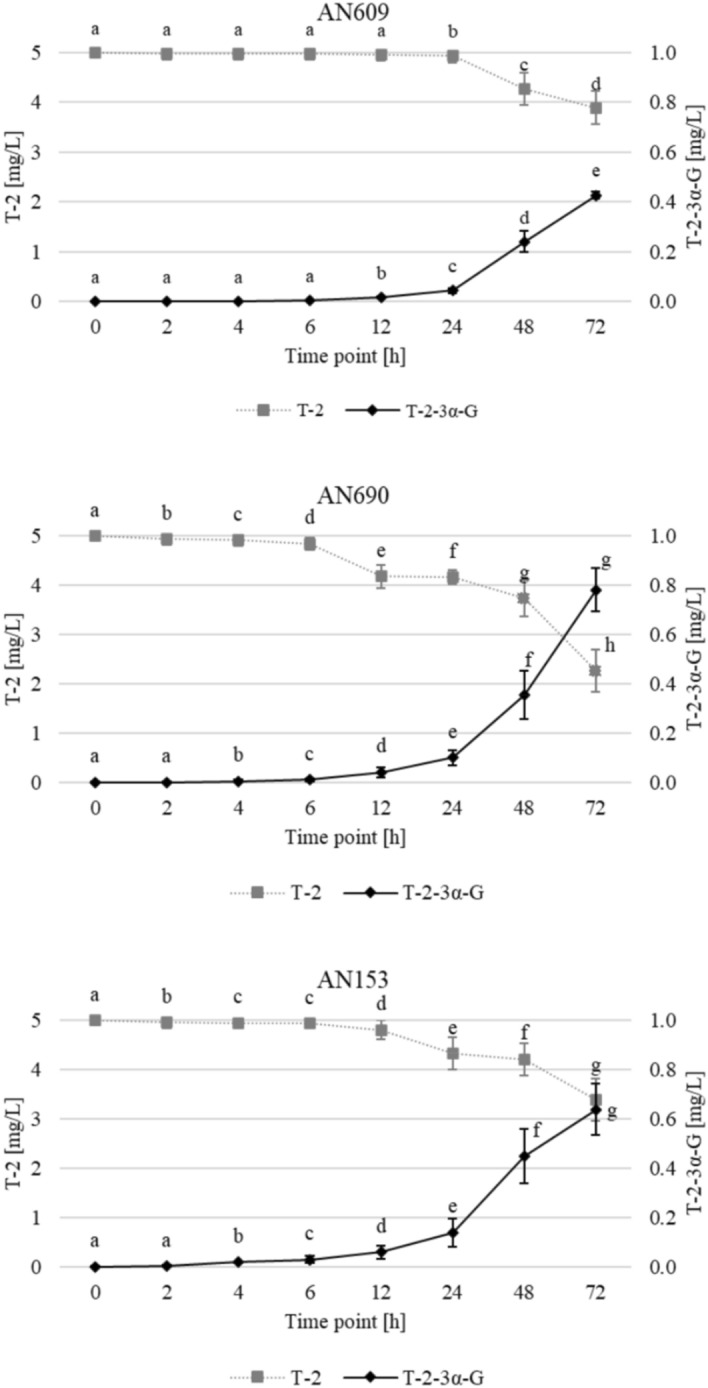
Changes in T-2 and T2-3α-G concentration during incubation experiments with selected isolates—AN609 (*T. viridescens)*, AN690 (*T. viride*), AN153 (*T*. *atroviride)*. Points with the same letter are not significantly different according to α = 0.01.

The above analyses allowed the selection of three isolates, each exemplifying a separate species within the *Trichoderma* genus: *T. atroviride* AN153*, T. viride* AN690, and *T. viridescens* AN609. The ability of these *Trichoderma* isolates to biotransformation T-2 toxin to its glucoside derivative (T2-3α-G) was investigated (Fig. 5; Figure S3). However, deacetylation of T-2 toxin to HT-2 and its glucosidic forms has not been reported.

Statistical analysis reveals significant differences between time points. It is observed that strain AN690 exhibits the highest biotransformation activity, as evidenced by a steady and significant increase in the T2-3α-G concentration, reaching the peak value among the strains. This strain demonstrates a rapid response in the initial hours with a continued rise until 72 h, suggesting a potent enzymatic activity towards the detoxification of T-2 toxin. In contrast, strain AN609 shows the least efficient biotransformation of T-2 toxin to T2-3α-G, with a much slower rate of increase in the T2-3α-G concentration. The content of the T-2 toxin correspondingly decreases for all strains, with AN690 showing the most pronounced reduction, aligning with the high glucoside area observed.

## Discussion

Co-culture assay on agar medium allowed the evaluation of the inhibitory effect of *Trichoderma* on the mycelial growth of *F. sporotrichioides* 2006a. All *Trichoderma* isolates showed an antagonistic effect against the pathogen, with *T. atroviride* strains (AN153, AN215, AN523, and AN705) revealing a stronger inhibitory effect (inhibition rate 32–49%). The limitation of the growth of pathogenic *Fusarium* species by *Trichoderma* has so far been evaluated by several authors^10,16,17^. The effect has been assessed on a number of species, hence the varying degree of growth inhibition of the pathogen. Relatively high pathogen growth inhibition values of the same species were obtained by Tian et al^10^., who showed that *T. harzianum* and *T. asperellum* inhibit the mycelial growth of *F. sporotrichioides *in vitro by up to 79%. Similar results to ours were obtained by Błaszczyk et al^16^., who observed that under in vitro conditions, *T. atroviride*, *T. viride*, and *T. viridescens* reduced the growth of *F. avenaceum*, *F. cerealis*, *F. culmorum*, *F. graminearum* and *F. temperatum* and the inhibition rate varied from 16 to 40% (depending on the strain). In contrast, Sallam et al^17^. showed that *Trichoderma* inhibit *F. oxysporum* mycelial growth by 47–68%, with the highest inhibition rates attributed to *T. atroviride* and *T. longibrachiatum* (59 and 68%, respectively)^17^. These results suggest that *Trichoderma* fungi strongly inhibit the growth of *Fusarium* and their potential is very likely related to the competition for nutrients and space. Similar observations were noted by Veenstra et al^18^. and Larran et al^7^.

Metabolomic studies have shown that there is a group of substances produced by fungi whose presence in the medium results from of co-culture (i.e. presence of individual substances may be the result of a competitor's growth stress and the presence of these metabolites was not observed in control media). Unfortunately, it is not possible to attribute individual metabolites to a particular microorganism, but it can be concluded with high probability that a significant proportion of these metabolites are the result of *Trichoderma* fungal growth, as these species were dominant during growth. Furthermore, of all the metabolites resulting from the co-culture of *Fusarium* and *Trichoderma* strains, the abundance of 49 of these compounds attributed to the *Trichoderma* fungi increased relative to the control samples (*Fusarium*-specific only one). The competitor may contribute to the enhanced inhibition of the biosynthesis of certain metabolites or the presence of metabolites not observed in the control strains. Until now the presence of metabolites that are a specific effect of fungal co-incubation has not been confirmed, making the discussion much more difficult^19–21^. *Trichoderma* is known to biosynthesize secondary metabolites that exhibit antifungal activity against *Fusarium*. Yassin et al^19^. found that the antagonistic potential of *T. harzianum* and *T. viride* against *F. proliferatum* and *F. verticillioides* is related to their ability to biosynthesize in vitro a number of bioactive components, such as acetic acid, harzianic acid, 6-pentyl-α-pyrone and 2H-pyran-2-one, 2-phenylethyl alcohol, dihydroxyacetone, hexadecanoic acid, and 9-eicosane. In contrast, Mironenka et al^20^. found that certain metabolites biosynthesized by *T. harzianum* (i.e. 14-aminoacids peptaibols, T-22-azophilone and harzianic acid) inhibit the growth of *F. culmorum *in vitro. There are reports that trichothecenes biosynthesized by some *Trichoderma* strains inhibit the growth of *Fusarium*. Shi et al^21^. showed that trichodermines biosynthesised by *T. brevicompactum* exhibit antifungal properties against *F. oxysporum*. Our study showed that metabolites that were not identified in the control media were present in the co-culture. Therefore, we suppose that the presence of these metabolites in the medium of the co-cultures may be the result of the transformation of compounds characteristic of one microorganism by the enzymatic system of the other. Tian et al^22^. observed that altenariol (AOH), *Alternaria* metabolite, can be biotransformed by *T. atroviride* via hydroxylation (AOH-OH), but the detailed mechanism has not yet been investigated. In addition, we observed the possibility of biotransformation of HT-2 and T-2 toxins, as partially confirmed by tests on liquid medium. The mechanism was based on glycosylation of HT-2 and T-2 toxins. The glucosides of HT-2 and T-2 toxins were not identified in the media of *Fusarium* monoculture, while they were present in the medium of co-cultures. *Fusarium sporotrichioides* isolates can biosynthesize glucosides of T-2 and HT-2 toxins on rice grain in vitro^23^. However, control *F. sporotrichoides* samples did not confirm it. So far, the ability of *Trichoderma* fungi to biotransform HT-2 and T-2 toxins was postulated by Tian et al^10^. Although, the presence of glucosides in the medium was confirmed only qualitatively. Depending on the *Trichoderma* strain (*T. harzianum*, *T. koningii*, *T. atroviride*, *T. asperellum*, *T. virens*), the degree of T-2 and HT-2 toxin biosynthesis reduction by *F. sporotrichioides* ranged from 12 to 82%^10^. A similar mechanism was confirmed against deoxynivalenol. Tian et al^24^. showed that when competing with *F. graminearum *in vitro*, Trichoderma* strains could biotransform deoxynivalenol to deoxynivalenol-3-glucoside. Also, Modrzewska et al^25^. suggested that the presence of deoxynivalenol-3-glucoside in the co-culture of *F. culmorum* and *Trichoderma* may result from of the antagonist's effect on the pathogen. In another experiment, Tian et al^26^. examined the ability of *Trichoderma* fungi to metabolize zearalenone, but studied strains did not show the ability to biotransform via glucosylation. There are reports that *Blastobotrys* fungi biotransform T-2 toxin to its glucosidic forms through the biosynthesis of glucosyltransferases^27^.

The phenomenon of glucosylation has been widely described in the context of mycotoxin detoxification by plants^28–30^ and our current and previous studies demonstrate that *Trichoderma*, like plants, may have the ability to detoxify trichothecenes^25^. In co-cultures, the presence of T-2-3α-G, HT-2-3α-G, and HT-2-3β-G was detected, whereas the T-2-3β-G form was not observed. It is particularly significant to highlight that T-2-3α-G was also identified in monocultures of *Trichoderma* that had been exposed to the T-2 toxin. This observation underscores the capability of *Trichoderma* to perform glycosylation, as the glucoside derivative was not initially present but emerged over time as a result of the biotransformation process. Similar observations were noted by McCormick et al^28^., where only T-2-3α-G was detected in naturally contaminated samples of oats and wheat. In the case of HT-2, both α and β anomers were present. Meanwhile, Meng-Reiterer et al^29^. identified only T-2-3β-G and HT-2-3β-G in barley spikes. These results point to the potential differentiation of metabolic pathways by various organisms to specifically detoxify mycotoxins. Thus, *Trichoderma* and plants may possess distinct enzymes or operate under varied environmental conditions, which may result in the preferential formation of specific anomers. In particular, the diversity of enzyme specificity, such as that of UDP-glucosyltransferases, may contribute to a preference for the synthesis of α-glucosides over β-glucosides^30^. The observed predominance of the α anomer for the T-2 toxin suggests a higher selectivity or activity of the enzymes involved in its modification.

To assess the effect of *Trichoderma* on the growth of *F. sporotrichioides* in co-cultures on rice, which may affect the production/content of the tested mycotoxins, the quantification of the pathogen’s DNA was undertaken in a single culture and in co-culture with *Trichoderma* by using digital PCR technology. This approach allowed for the estimation of the percentage of reduction in the pathogen’s DNA content in co-cultures on rice, which may indirectly suggest a reduction in the growth of *F. sporotrichioides* in the presence of *Trichoderma*. Consequently, these data may influence conclusions regarding the content of mycotoxins in the post-culture samples tested. Based on the analyzes performed, it can be concluded that the highest concentrations of toxins were found in samples from the coculture of *F. sporotrichioides* and *T. atroviride* AN215, in which the lowest percentage of reduction in the pathogen’s DNA content was recorded. It is therefore assumed that the higher content of mycotoxins in these samples may be related to the higher content of *F. sporotrichioides* mycelium than in the other co-cultures. Meanwhile, lower concentrations of toxins were detected in post-culture samples in which the *F. sporotrichioides* DNA content was reduced by over 90%.

The detection of unique metabolites in co-cultures could indicate the modification of metabolic pathways by one organism in response to the presence of a competing species. Biosynthesis of trichothecenes by *F. sporotrichioides*, particularly in the context of existing enzymatic pathways, underscores the complex nature of these processes^31^. Noteworthy is the presence of metabolites with fragment ions m/z 249.113 and m/z 231.102 in co-culture which according to Cui et al^32^. are characteristic for type B trichothecenes. For *F. sporotrichioides* the typical pathway does not lead to the formation of type B trichothecenes^31^. The presence of *Trichoderma* in the co-culture can activate pathways in *Fusarium* associated with stress response, such as oxidative stress or response to cellular damage. Such changes can influence metabolism and lead to alterations in trichothecene biosynthesis^33^. Stress induced by *Trichoderma* may lead to the reorganization of metabolic priorities in *Fusarium*, with possible activation of trichothecene biosynthesis pathways as part of a broader defensive response^34^. This might also explain the presence of diacetoxyscirpenol (DAS) derivatives in the co-cultures. The biosynthesis of trichothecenes by *Fusarium* in response to *Trichoderma* could be similar to the production of these toxins in response to other environmental stressors, such as the presence of other microorganisms, mechanical damage, or changes in nutrient availability^35^.

In our study, we demonstrated that the tested *Trichoderma* isolates exhibit antagonistic activity against *F. sporotrichioides* 2006a, with inhibition levels dependent on the specific species and strain of *Trichoderma*. Metabolomic studies revealed novel substances in the co-culture medium, absent in controls, likely secreted due to stress from the presence of a competitor. Additionally, *Trichoderma* fungi not only showed antagonism towards pathogenic fungi but also inhibited the biosynthesis and potentially transformed T-2 and HT-2 toxins via glucosylation. These results highlight *Trichoderma* as biocontrol agents and emphasize the importance of further studies to elucidate the processes involved in mycotoxin detoxification.

## Materials and methods

### Fungal strains

*Trichoderma* strains used in the study: *T. atroviride* AN153, *T. atroviride* AN215, *T. atroviride* AN523, *T. atroviride* AN705, *T. viride* AN355, *T. viride* AN690, *T. viridescens* AN508, *T. viridescens* AN609 and the strain *F. sporotrichioides* KF 2006a (throughout the article described as 2006a) were obtained from the collection of the Institute of Plant Genetics of the Polish Academy of Sciences in Poznań, Poland.

### Chemicals and reagents

Analytical standards for HT-2 and T-2 toxins were purchased from Romer Labs (Tulln, Austria). The standards of α and β anomers of T-2-3-glucoside (T-2-3α-G and T-2-3β-G) and HT-2-3-glucoside (HT-2-3α-G and HT-2-3β-G) were synthesized according to Schmidt et al^36^. T-2-3α-G was produce via biotransformation by the yeast *Blastobotrys muscicola* according to McCormick et al^27^. LC–MS grade methanol, acetonitrile, and water were purchased from Witko (Lodz, Poland). LC–MS grade formic acid and ammonium formate were purchased from Chem-Lab (Zedelgem, Belgium). Fungal culture was carried out on potato-dextrose agar (PDA) medium (Oxoid, Basingstoke, UK), in liquid Czapek-Dox medium (Sigma-Aldrich, Merck KGaA, Darmstadt, Germany) and white rice from the market.

### Direct antagonism assay by the co-culture of *Trichoderma* fungi and *F. sporotrichioides*

Mycelial discs of 2 mm-diameter from the 7-day revitalized cultures on potato dextrose agar (PDA, Sigma Aldrich, Darmstadt, Germany) were taken and transferred to PDA medium in a combination of each antagonist isolate with a pathogen on opposite sides of a Petri dish (85 mm diameter). The controls were grown in monocultures. Cultures were incubated at 25 ± 2 °C, 12 h/12 h night/day for 5 days. Each culture was performed in three replicates. The radial growth of the colony size was measured with a ruler every 24 h, and measurements were used to calculate the colony growth rate (mm/day). Inhibitory effect of antagonists on pathogen was estimated as the percentage reduction in pathogen growth in the presence of the antagonist, in accordance with the formula: (Rc − R)/Rc × 100 where R_c_ is the pathogen's mycelial growth in the control, and R is the pathogen's mycelial growth in the co-culture^16^.

### Fusarium mycotoxins analysis and metabolomic analysis

#### Cultivation on solid substrate

Fungi were inoculated on rice in combinations of each antagonist isolate and a pathogen isolate (TF). Isolates growing in single cultures (T—*Trichoderma* strains, F—*F. sporotrichioides* 2006a) were the controls. Solid state assay on rice were prepared according to the method described by Modrzewska et al^25^. Sterile rice grain was inoculated with 3 cm^2^ of mycelial surface grown on PDA, and this procedure was replicated three times to ensure consistency and reliability in the results. After a 14-day incubation, the cultures were dried at room temperature and ground using a knife mill (Grindomix GM 200, Retsch, Haan, Germany). Samples were prepared according to the modified method described by Uwineza et al^37^. Crushed rice samples (0.5 g) were extracted with 20 mL of a mixture of acetonitrile: 0.1% HCOOH_aq_ (84:16, v/v) using a homogenizer (Unidrive X 1000, Cat Scientific, Paso Robles, USA) at 5,000 rpm for 2 min. The mixture was then centrifuged for 10 min in a laboratory centrifuge (MPW-380R, MPW Med. Instruments, Warsaw, Poland) at 10,000 rpm. The resulting supernatant was filtered through a nylon 0.45 µm syringe filter, and 2 mL was transferred to a 5 mL-round-bottom flask and evaporated in a vacuum evaporator (Heidolph Instruments, Schwabach, Germany). The residue was dissolved with 1 mL of a mixture of methanol: 0.25% HCOOH*aq* (60:40, v/v), sonicated for 2 min and filtered through a nylon 0.22 µm syringe filter. Then, the samples were used for quantification of mycotoxins, metabolomic analysis and percentage of the *F. sporotrichioides* 2006a mycelium growth reduction by *Trichoderma* strains.

#### Cultivation on liquid substrate

Mycelial discs of 3 mm-diameter from the 7-day cultures of *F. sporotrichioides* 2006a and previously selected *Trichoderma* strains were used as an inoculum. 100 ml falcons filled with 25 mL Czapek-Dox broth medium (Sigma-Aldrich, Saint Louis, MI, USA) supplemented with yeast extract (10 g/L, Oxoid™ Yeast Extract Powder Thermo Fisher Scientific, Waltham, MA, USA) incubated at 25 °C in 100 rpm rotary shaking for 96 h. The T-2 toxin stock was added after 24 h of incubation. The initial concentration of T-2 toxin in the liquid cultures was 5 mg/L. The samples (both mycelium and medium) were collected before and after the addition of the toxin. The samples were collected after one minute (0), 2, 4, 6, 12, 24, 48 and 72 h after toxin application. After culture, the mycelium was centrifuged and placed at –30 °C for 24 h, then lyophilized in an ALPHA 1-4 LSCplus (Martin Christ Gefriertrocknungsanlagen GmbH., Osterode am Harz, Germany) unit for 24 h at 25 °C. The medium was diluted 1:10 with a mixture of acetonitrile: water (84:16, v/v), sonicated for 2 min and filtered through a nylon 0.22 µm syringe filter. The mycelium was extruded with 0.5 mL of the mixture of acetonitrile: water (84:16, v/v), sonicated for 30 min and filtered through a nylon 0.22 µm syringe filter. The collected material was used for qualitative metabolomic analysis.

#### Analysis of mycotoxins

Quantitative analysis of mycotoxins was carried out using a liquid chromatography-Q-Exactive Orbitrap mass spectrometer operating with a heated electrospray interface (UHPLC-HESI-MS/MS) in the parallel reaction monitoring mode (PRM) (Thermo Fisher Scientific, Waltham, MA, USA). The analytes were separated on a C18 Cortecs chromatography column (100 mm × 2.1 mm × 1.6 μm, Waters). For the quantitative analysis, the mobile phase consisted of water and methanol in ratios of 90:10 (phase A) and 10:90 (phase B), respectively. Both phases contained 5 mM ammonium formate and 0.2% formic acid. The following flow gradient was used: from 0 to 6 min 0% B; 6 to 29 min 0 to 40% B; 29 to 40 min 40 to 70% B; and 40 to 45 min 70 to 100% B. The flow rate was 0.3 mL/min, and the injection volume was 2.5 µL. The mass spectrometer was operated with resolution of 70,000 in positive–negative switching ionization mode, with a scan range of *m/z* 100 to 1,500. The parameters of the ion source were as follows: sputtering voltage 3.2 kV (for positive polarity) and 2.2 kV (for negative polarity), HESI temperature 350 °C, shielding gas pressure 40 IU, auxiliary gas pressure 10 IU, and ion transfer tube temperature 250 °C. Parameters specific to the identification of the analytes under study are tabulated (Table 4S).

#### Metabolomic analysis and data processing

The metabolic profiling was performed using Vanquish UHPLC unit hyphenated with Exploris 120 Orbitrap mass spectrometer (Thermo Fisher Scientific, Waltham, MA, USA). Water (phase A) and acetonitrile: water (20:80) mixture (phase B) were used as mobile phases. Both phases contained the addition of 5 mM ammonium formate and 0.2% formic acid. The following flow gradient was used: from 0 to 2 min 0% B; 2 to 55 min 0 to 100% B; 55 to 56 min 100% B. The ion source parameters and scan range were the same as in the quantitative analysis. Data were acquired in the full-scan MS/data-dependent MS2 (ddMS2) mode. MS cycles were composed of 1 full scan and 4 ddMS2 scans in a particular ionization. The four ions with the most intense signal detected in the full MS survey scan (intensity threshold 4.0 × 10^4^) triggered an MS2 event at the peak apex with an isolation window of *m/z* 1.5. A 2.0 s delay was required for the same ion to trigger a new MS2 event (dynamic exclusion). A relatively short dynamic exclusion bracket was selected so that MS2 fragmentation data could be generated for closely eluting isobaric compounds. Full MS scans were acquired from *m/z* 100 to 1500 with a resolution of 120,000. Fragmentary ion scans (ddMS2) were acquired at a resolution of 15,000. Ions were fragmented with stepped collision energy 10 and 40% (normalized). The resulting data were processed in Compound Discoverer™ (version 3.3; Thermo Scientific, Fremont, CA, USA) to generate a list of potential metabolites. The data was processed twice: with no peak intensity threshold and with one set to 5.0 × 10^5^. The Expected Compounds’ nodes were used to generate a mass list of possible metabolites of T-2, HT-2, neosolaniol, and diacetoxyscirpenol.

### Analysis of *F. sporotrichioides* growth inhibition on rice solid substrate by *Trichoderma*

The percentage of *F. sporotrichioides* growth inhibition by *Trichoderma* strains was estimated based on the quantification of the pathogen's DNA using the QIAcuity EG PCR Kit (Qiagen, Hilden, Germany) with the dsDNA-binding dye EvaGreen® in a singleplex reaction using QIAGEN’s QIAcuity-02411 (Qiagen, Hilden, Germany) instruments for digital PCR (dPCR). For dPCR assay, DNA was isolated from 50 mg solid substrate culture samples ground to a power using the Wizard® Genomic DNA Purification Kit (Promega, Madison, WI, USA). The obtained total DNA was quantitatively and qualitatively assessed using Nanodrope and was initially used to confirm the specificity and sensitivity of PCR and dPCR tests by optimizing the reaction, including the concentration of DNA and primers and their hybridization temperature. The primers specific to the species *F. sporotrichioides* were used in the analyses: FspoA18 fwd (5′–GCAAGTCGACCACTGTGAGTACA–3′) and FspoA85 rev (5′–CTGTCAAAGCATGTCAGTAAAAATGAT–3′)^38^. The reaction mix with a total volume of 40 μL was composed of 13.3 μL of 3 × EvaGreen PCR Master Mix (QIAcuity EG PCR Kit, Qiagen, Hilden, Germany), 4 μL of 10 × primer mix (each of a pair with a concentration of μM), 21.7 μL RNase -free water and 1 μL of DNA template. Several variants were used as template DNA. The first variant included: (a) DNA mix from all *Trichoderma* strains in co-cultures on rice with *Fusarium* samples; (b) DNA mix from all *Fusarium* in a single culture on rice samples; (c) DNA mix from all *Trichoderma* in a single culture on rice samples used as a negative control; (d) RNase -free water used as a non-template control (NTC). The second variant included: (a) DNA mix from 3 biological replicates for each *Trichoderma* strain in co-culture on rice with *Fusarium* samples; (b) DNA mix from 3 biological replicates for *Fusarium* in a single culture on rice samples; (c) DNA mix from all *Trichoderma* in single cultures on rice samples used as a negative control; (d) RNase -free water used as a non-template control. The reaction mixtures were loaded onto Nanoplate 26 k (24-well, (Qiagen, Hilden, Germany) and placed in the instrument QIAGEN’s QIAcuity-02411 (Qiagen, Hilden, Germany). The following dPCR steps were used: (1) QIAGEN Standard Priming Profile; (2) cycling profile (95 °C for 2 min followed by 40 cycles of 95 °C for 15 s, 58 °C for 15 s, 72 °C for 15 s, and cooling to 40 °C for 5 min); (3) imaging profile (channel green, 500 ms exposure duration, gain 6) The dPCR results were analyzed using QIAcuity Software Suite 2.2.0.26 (Qiagen, Hilden, Germany). The percentage of DNA concentration reduction in co-cultures on solid rice substrate was estimated according to the formula: (DNA-F_c_–DNA-F/T)/ DNA-F_c_ × 100 where DNA-F_c_ is the pathogen's DNA concentration in the control, and DNA-F/T is the pathogen's DNA concentration in the co-culture.

### Data analysis

Statistical analysis was performed using Statistica 13.1 software (StatSoft, Poland). One-way analysis of variance (One Way Anova) was used to determine the significance of pathogen growth inhibition by *Trichoderma*, the degree of inhibition of toxin biosynthesis and change in peak area during incubation experiments with selected isolates. The homogeneity of groups was determined by Tukey’s HSD test. The significance of differences was determined at α = 0.01 level. The metabolomic data obtained were processed by filtering the peak area ratios and significance (*p*-value) between groups. The results were compiled and visualized (PCA analysis) using the Compound Discoverer 3.3 software.

### Supplementary Information

Supplementary Information.

## Data Availability

The datasets used and/or analysed during the current study available from the corresponding author on reasonable request.
