# Supplementary material for: Antagonistic properties against Fusarium sporotrichioides and glycosylation of HT-2 and T-2 toxins by selected Trichoderma strains
Source: Sci Rep. 2024 Mar 11;14:5865. doi: 10.1038/s41598-024-55920-x (PMC10928170; doi:10.1038/s41598-024-55920-x)
Supplement: Supplementary file 1 — Supplementary Information. [file 41598_2024_55920_MOESM1_ESM.docx]

**Antagonistic properties against *Fusarium sporotrichioides* and glycosylation of HT-2 and T-2 toxins by selected *Trichoderma* strains**

Marta Modrzewska^1^, Dominik Popowski^1^, Lidia Błaszczyk^2^, Łukasz Stępień^3^, Monika Urbaniak^3^, Marcin Bryła^1^, Benedikt Cramer^4^, Hans-Ulrich Humpf^4^, Magdalena Twarużek^5^

^1^Department of Food Safety and Chemical Analysis, Prof. Waclaw Dabrowski Institute of Agricultural and Food Biotechnology - State Research Institute, Rakowiecka 36, 02-532 Warsaw, Poland

^2^Plant Microbiomics Team, Institute of Plant Genetics, Polish Academy of Sciences, 60-479 Poznan, Poland

^3^Plant-Pathogen Interaction Team, Institute of Plant Genetics, Polish Academy of Sciences, 60-479 Poznan, Poland

^4^Institute of Food Chemistry, University of Münster, Corrensstr. 45, 48149, Münster, Germany

^5^Kazimierz Wielki University, Faculty of Natural Sciences, Institute of Experimental Biology, Department of Physiology and Toxicology, Chodkiewicza 30, 85–064 Bydgoszcz, Poland

**Corresponding author:** Marcin Bryła, Email: marcin.bryla@ibprs.pl

**Supplementary Material**

**Table 1S.** The list of metabolites with peak area ratio ≥ 5 and p-value ≤ 0.05 in one of the groups. F: *Fusarium* monoculture, T: *Trichoderma* monoculture, TF: co-culture.

| **#** | **m/z** | **RT [min]** | **Ratio** | **P- value** | **Ratio** | **P- value** |
| --- | --- | --- | --- | --- | --- | --- |
|  |  |  | **(TF)/(F)** | **(TF)/(T)** | **(TF)/(F)** | **(TF)/(T)** |
| 1 | 153.0548 | 6.758 | 9.303 | 0.021 | 5.539 | 0.004 |
| 2 | 163.0755 | 6.81 | 11.706 | 0.005 | 8.586 | 0.019 |
| 3 | 171.1858 | 19.567 | 24.101 | 0.014 | 19.207 | 0.017 |
| 4 | 188.1647 | 15.096 | 16.663 | 0.050 | 10.681 | 0.019 |
| 5 | 188.1647 | 11.884 | 30.628 | 0.006 | 15.234 | 0.005 |
| 6 | 188.1648 | 12.21 | 9.228 | 0.003 | 8.183 | 0.022 |
| 7 | 191.1069 | 10.775 | 14.292 | 0.015 | 14.873 | 0.014 |
| 8 | 195.0656 | 8.54 | 12.407 | 0.001 | 9.929 | 0.000 |
| 9 | 195.0744 | 2.329 | 10.742 | 0.014 | 5.663 | 0.036 |
| 10 | 195.1746 | 24.192 | 5.749 | 0.037 | 5.937 | 0.030 |
| 11 | 196.1699 | 14.363 | 6.062 | 0.002 | 6.128 | 0.016 |
| 12 | 197.0811 | 6.855 | 6.527 | 0.000 | 5.229 | 0.001 |
| 13 | 211.0281 | 6.332 | 13.873 | 0.005 | 9.772 | 0.004 |
| 14 | 213.0761 | 7.975 | 7.251 | 0.013 | 5.711 | 0.015 |
| 15 | 213.0761 | 6.984 | 7.169 | 0.035 | 6.697 | 0.044 |
| 16 | 216.0998 | 11.526 | 17.941 | 0.001 | 13.498 | 0.002 |
| 17 | 222.1161 | 8.764 | 8.010 | 0.009 | 7.248 | 0.009 |
| 18 | 224.1033 | 8.506 | 21.372 | 0.001 | 5.392 | 0.001 |
| 19 | 224.2013 | 31.134 | 11.404 | 0.001 | 9.082 | 0.014 |
| 20 | 225.0761 | 7.575 | 6.710 | 0.004 | 7.080 | 0.004 |
| 21 | 232.0948 | 9.326 | 8.254 | 0.003 | 7.191 | 0.014 |
| 22 | 232.1548 | 8.687 | 11.171 | 0.003 | 6.486 | 0.005 |
| 23 | 234.044 | 6.339 | 10.186 | 0.006 | 6.415 | 0.018 |
| 24 | 234.0442 | 6.331 | 9.268 | 0.007 | 5.942 | 0.018 |
| 25 | 238.2169 | 33.933 | 8.294 | 0.013 | 5.255 | 0.003 |
| 26 | 240.1234 | 10.268 | 13.307 | 0.005 | 6.217 | 0.022 |
| 27 | 243.1706 | 7.957 | 78.488 | 0.023 | 5.875 | 0.007 |
| 28 | 249.0606 | 1.203 | 11.858 | 0.004 | 5.926 | 0.046 |
| 29 | 251.1603 | 0.666 | 35.825 | 0.019 | 8.955 | 0.026 |
| 30 | 251.1604 | 0.67 | 22.343 | 0.024 | 7.246 | 0.044 |
| 31 | 252.1236 | 1.972 | 10.594 | 0.000 | 7.481 | 0.007 |
| 32 | 253.1072 | 12.56 | 8.775 | 0.004 | 8.353 | 0.003 |
| 33 | 253.1775 | 18.31 | 7.057 | 0.002 | 6.081 | 0.038 |
| 34 | 257.1573 | 20.635 | 14.518 | 0.001 | 7.488 | 0.045 |
| 35 | 259.1655 | 7.927 | 25.586 | 0.003 | 8.608 | 0.031 |
| 36 | 260.1609 | 6.028 | 11.394 | 0.009 | 7.182 | 0.045 |
| 37 | 261.0872 | 10.264 | 19.433 | 0.005 | 7.139 | 0.037 |
| 38 | 267.136 | 16.716 | 5.442 | 0.029 | 5.017 | 0.018 |
| 39 | 268.0817 | 1.049 | 8.383 | 0.003 | 7.153 | 0.038 |
| 40 | 268.1295 | 8.558 | 9.620 | 0.003 | 6.337 | 0.000 |
| 41 | 273.1449 | 1.078 | 15.334 | 0.000 | 15.556 | 0.007 |
| 42 | 276.1084 | 3.244 | 5.486 | 0.002 | 5.019 | 0.002 |
| 43 | 281.1387 | 20.445 | 8.106 | 0.022 | 6.494 | 0.042 |
| 44 | 286.1445 | 3.152 | 10.920 | 0.001 | 9.546 | 0.001 |
| 45 | 288.1234 | 13.369 | 8.932 | 0.035 | 8.118 | 0.044 |
| 46 | 288.1921 | 7.698 | 7.327 | 0.003 | 5.797 | 0.033 |
| 47 | 288.1922 | 6.326 | 9.328 | 0.007 | 6.914 | 0.010 |
| 48 | 289.1186 | 6.433 | 14.483 | 0.018 | 5.314 | 0.030 |
| 49 | 290.1025 | 9.19 | 11.749 | 0.002 | 10.258 | 0.010 |
| 50 | 290.1026 | 9.201 | 10.370 | 0.002 | 8.866 | 0.007 |
| 51 | 293.2088 | 23.332 | 10.089 | 0.003 | 9.464 | 0.008 |
| 52 | 300.16 | 7.605 | 7.320 | 0.008 | 7.296 | 0.006 |
| 53 | 300.1921 | 6.637 | 15.352 | 0.003 | 9.168 | 0.001 |
| 54 | 305.1387 | 23.251 | 8.038 | 0.008 | 7.786 | 0.009 |
| 55 | 307.1883 | 25.975 | 8.568 | 0.008 | 7.861 | 0.008 |
| 56 | 311.1606 | 10.11 | 8.386 | 0.009 | 5.154 | 0.006 |
| 57 | 311.1625 | 13.118 | 5.383 | 0.012 | 6.013 | 0.004 |
| 58 | 316.1161 | 4.287 | 13.564 | 0.003 | 9.167 | 0.010 |
| 59 | 318.1664 | 6.513 | 12.442 | 0.001 | 5.370 | 0.025 |
| 60 | 318.2643 | 24.182 | 6.904 | 0.033 | 6.725 | 0.034 |
| 61 | 320.1972 | 12.352 | 10.496 | 0.026 | 7.416 | 0.008 |
| 62 | 322.9755 | 6.324 | 14.535 | 0.007 | 9.225 | 0.022 |
| 63 | 332.0901 | 4.308 | 7.329 | 0.006 | 6.277 | 0.006 |
| 64 | 332.2183 | 7.578 | 5.551 | 0.035 | 5.088 | 0.024 |
| 65 | 332.2184 | 10.363 | 8.953 | 0.000 | 8.070 | 0.003 |
| 66 | 332.2184 | 12.575 | 12.682 | 0.000 | 6.522 | 0.013 |
| 67 | 336.1922 | 12.47 | 6.882 | 0.016 | 9.287 | 0.005 |
| 68 | 336.1925 | 12.466 | 6.692 | 0.008 | 6.460 | 0.001 |
| 69 | 342.239 | 13.012 | 17.155 | 0.001 | 5.862 | 0.003 |
| 70 | 342.2391 | 12.818 | 11.602 | 0.005 | 5.053 | 0.021 |
| 71 | 344.2546 | 12.697 | 8.829 | 0.004 | 5.089 | 0.027 |
| 72 | 344.2548 | 13.831 | 15.346 | 0.003 | 6.346 | 0.001 |
| 73 | 345.2317 | 12.776 | 19.094 | 0.000 | 5.600 | 0.017 |
| 74 | 346.234 | 12.764 | 17.135 | 0.000 | 5.668 | 0.015 |
| 75 | 347.1969 | 14.158 | 7.747 | 0.002 | 5.611 | 0.050 |
| 76 | 349.1764 | 8.522 | 9.386 | 0.006 | 8.532 | 0.005 |
| 77 | 351.1917 | 8.529 | 9.954 | 0.036 | 9.053 | 0.039 |
| 78 | 355.1867 | 9.91 | 105.134 | 0.017 | 7.486 | 0.024 |
| 79 | 360.2133 | 10.441 | 7.290 | 0.000 | 5.880 | 0.011 |
| 80 | 362.2077 | 12.003 | 7.489 | 0.022 | 7.244 | 0.010 |
| 81 | 362.208 | 11.999 | 13.444 | 0.014 | 8.933 | 0.010 |
| 82 | 363.8897 | 12.404 | 15.261 | 0.002 | 7.579 | 0.004 |
| 83 | 364.2235 | 12.414 | 13.008 | 0.002 | 5.695 | 0.018 |
| 84 | 366.101 | 8.83 | 133.152 | 0.005 | 8.885 | 0.014 |
| 85 | 366.2028 | 14.167 | 9.074 | 0.000 | 7.687 | 0.002 |
| 86 | 372.1139 | 1.032 | 9.150 | 0.017 | 5.084 | 0.046 |
| 87 | 372.1899 | 9.157 | 233.508 | 0.007 | 13.489 | 0.031 |
| 88 | 372.213 | 7.363 | 37.858 | 0.004 | 14.762 | 0.014 |
| 89 | 372.2132 | 7.906 | 110.305 | 0.005 | 20.078 | 0.007 |
| 90 | 372.2134 | 7.349 | 34.496 | 0.004 | 15.261 | 0.013 |
| 91 | 373.1973 | 9.155 | 414.524 | 0.000 | 15.310 | 0.033 |
| 92 | 374.229 | 12.918 | 6.703 | 0.022 | 5.129 | 0.001 |
| 93 | 375.1736 | 10.969 | 40.937 | 0.001 | 13.884 | 0.012 |
| 94 | 377.0692 | 1.029 | 6.458 | 0.029 | 6.356 | 0.037 |
| 95 | 378.2392 | 14.07 | 7.538 | 0.000 | 6.182 | 0.001 |
| 96 | 383.1709 | 10.903 | 72.091 | 0.006 | 9.654 | 0.030 |
| 97 | 388.2084 | 7.281 | 45.371 | 0.002 | 21.633 | 0.007 |
| 98 | 389.2115 | 7.307 | 6.858 | 0.010 | 7.572 | 0.020 |
| 99 | 390.9631 | 6.331 | 8.114 | 0.004 | 6.377 | 0.008 |
| 100 | 394.2341 | 12.011 | 6.244 | 0.000 | 5.348 | 0.019 |
| 101 | 395.1602 | 25.998 | 31.404 | 0.014 | 9.602 | 0.018 |
| 102 | 397.1974 | 14.344 | 5.164 | 0.017 | 6.231 | 0.016 |
| 103 | 401.1745 | 11.264 | 41.319 | 0.012 | 14.326 | 0.021 |
| 104 | 401.7327 | 11.471 | 7.745 | 0.018 | 5.195 | 0.045 |
| 105 | 402.1444 | 10.91 | 24.691 | 0.025 | 5.994 | 0.023 |
| 106 | 402.1444 | 10.901 | 23.184 | 0.016 | 7.683 | 0.016 |
| 107 | 402.2241 | 8.902 | 7.068 | 0.021 | 6.509 | 0.009 |
| 108 | 414.2239 | 10.65 | 31.171 | 0.004 | 14.918 | 0.012 |
| 109 | 414.2604 | 12.036 | 7.912 | 0.004 | 5.269 | 0.011 |
| 110 | 414.2604 | 12.228 | 8.234 | 0.015 | 7.436 | 0.017 |
| 111 | 423.1226 | 9.369 | 27.182 | 0.007 | 31.018 | 0.006 |
| 112 | 423.2243 | 10.815 | 10.197 | 0.006 | 5.103 | 0.011 |
| 113 | 423.2244 | 10.802 | 11.116 | 0.003 | 5.103 | 0.015 |
| 114 | 430.2552 | 12.092 | 88.388 | 0.001 | 31.145 | 0.046 |
| 115 | 430.2552 | 12.09 | 92.291 | 0.001 | 31.422 | 0.046 |
| 116 | 438.2064 | 12.572 | 9.452 | 0.000 | 5.640 | 0.003 |
| 117 | 444.1206 | 10.909 | 11.724 | 0.019 | 5.893 | 0.003 |
| 118 | 444.2346 | 11.742 | 33.145 | 0.049 | 13.967 | 0.007 |
| 119 | 445.3682 | 45.171 | 9.188 | 0.005 | 5.440 | 0.005 |
| 120 | 446.2503 | 9.075 | 6.596 | 0.002 | 5.192 | 0.001 |
| 121 | 450.0538 | 8.834 | 8.605 | 0.025 | 6.083 | 0.026 |
| 122 | 457.2662 | 8.813 | 8.501 | 0.009 | 5.783 | 0.023 |
| 123 | 459.2728 | 30.802 | 6.311 | 0.008 | 7.646 | 0.009 |
| 124 | 467.2506 | 9.431 | 40.052 | 0.001 | 5.374 | 0.027 |
| 125 | 473.2611 | 8.835 | 19.438 | 0.001 | 13.604 | 0.014 |
| 126 | 474.2451 | 9.828 | 20.403 | 0.001 | 16.774 | 0.031 |
| 127 | 474.2918 | 15.015 | 5.912 | 0.003 | 6.380 | 0.004 |
| 128 | 480.1485 | 7.63 | 7.017 | 0.005 | 5.103 | 0.014 |
| 129 | 495.0969 | 9.155 | 5.907 | 0.024 | 5.148 | 0.019 |
| 130 | 502.2402 | 9.278 | 14.513 | 0.002 | 12.254 | 0.032 |
| 131 | 503.299 | 31.311 | 17.010 | 0.007 | 18.713 | 0.005 |
| 132 | 504.3264 | 31.871 | 6.997 | 0.006 | 5.183 | 0.022 |
| 133 | 516.2457 | 10.081 | 115.268 | 0.014 | 53.881 | 0.024 |
| 134 | 517.2508 | 10.026 | 43.495 | 0.008 | 10.298 | 0.011 |
| 135 | 517.2511 | 9.225 | 34.248 | 0.001 | 20.428 | 0.027 |
| 136 | 518.2615 | 11.017 | 22.950 | 0.023 | 8.008 | 0.019 |
| 137 | 518.3178 | 15.82 | 7.247 | 0.005 | 5.509 | 0.006 |
| 138 | 518.3183 | 15.825 | 6.574 | 0.000 | 6.086 | 0.001 |
| 139 | 543.1977 | 17.921 | 11.247 | 0.020 | 9.665 | 0.032 |
| 140 | 545.2235 | 10.792 | 14.683 | 0.043 | 12.379 | 0.001 |
| 141 | 546.2555 | 17.491 | 5.975 | 0.000 | 5.407 | 0.000 |
| 142 | 547.3254 | 31.747 | 16.754 | 0.003 | 14.789 | 0.004 |
| 143 | 551.2107 | 17.497 | 5.925 | 0.005 | 5.614 | 0.004 |
| 144 | 558.3496 | 21.382 | 6.607 | 0.015 | 5.349 | 0.007 |
| 145 | 562.3445 | 16.54 | 5.931 | 0.004 | 5.405 | 0.007 |
| 146 | 566.3191 | 10.067 | 37.880 | 0.008 | 5.244 | 0.034 |
| 147 | 567.2055 | 11.546 | 8.034 | 0.003 | 8.871 | 0.004 |
| 148 | 582.3142 | 9.873 | 27.285 | 0.007 | 10.505 | 0.030 |
| 149 | 582.3142 | 10.147 | 37.145 | 0.009 | 6.859 | 0.042 |
| 150 | 582.3142 | 10.021 | 23.438 | 0.009 | 9.656 | 0.010 |
| 151 | 591.3517 | 32.112 | 8.199 | 0.005 | 6.478 | 0.006 |
| 152 | 598.3091 | 10.074 | 160.301 | 0.000 | 7.364 | 0.003 |
| 153 | 598.3091 | 9.807 | 105.461 | 0.001 | 9.039 | 0.003 |
| 154 | 598.3098 | 9.797 | 125.152 | 0.001 | 9.039 | 0.003 |
| 155 | 601.3739 | 21.848 | 6.504 | 0.011 | 7.499 | 0.003 |
| 156 | 602.376 | 21.846 | 7.133 | 0.010 | 7.205 | 0.000 |
| 157 | 604.2974 | 20.244 | 16.702 | 0.002 | 18.229 | 0.000 |
| 158 | 609.2524 | 20.249 | 6.765 | 0.027 | 6.474 | 0.000 |
| 159 | 615.3353 | 10.909 | 23.592 | 0.018 | 6.562 | 0.028 |
| 160 | 615.3353 | 10.903 | 19.211 | 0.019 | 5.727 | 0.024 |
| 161 | 625.3198 | 9.536 | 9.626 | 0.003 | 8.688 | 0.023 |
| 162 | 638.3403 | 10.423 | 7.705 | 0.024 | 7.437 | 0.024 |
| 163 | 638.3405 | 10.421 | 7.292 | 0.025 | 7.038 | 0.026 |
| 164 | 646.3073 | 23.25 | 14.992 | 0.028 | 233.910 | 0.000 |
| 165 | 646.3077 | 23.249 | 14.992 | 0.028 | 217.306 | 0.000 |
| 166 | 646.4022 | 22.269 | 6.704 | 0.007 | 8.960 | 0.003 |
| 167 | 680.3414 | 15.237 | 6.455 | 0.002 | 5.450 | 0.018 |
| 168 | 690.4283 | 22.686 | 8.331 | 0.026 | 8.871 | 0.005 |
| 169 | 695.3979 | 11.022 | 252.383 | 0.003 | 8.895 | 0.018 |
| 170 | 709.3783 | 10.93 | 18.259 | 0.009 | 13.332 | 0.018 |
| 171 | 711.3927 | 10.967 | 654.530 | 0.000 | 15.980 | 0.014 |
| 172 | 712.743 | 23.242 | 9.205 | 0.001 | 9.125 | 0.007 |
| 173 | 713.2333 | 23.249 | 9.278 | 0.008 | 9.014 | 0.009 |
| 174 | 725.3722 | 11.732 | 33.889 | 0.028 | 21.630 | 0.004 |
| 175 | 725.3723 | 11.59 | 7.411 | 0.009 | 6.516 | 0.001 |
| 176 | 727.3868 | 10.903 | 923.432 | 0.007 | 8.931 | 0.032 |
| 177 | 727.3886 | 10.897 | 898.738 | 0.007 | 8.814 | 0.032 |
| 178 | 734.4546 | 23.051 | 10.733 | 0.041 | 9.404 | 0.002 |
| 179 | 737.3109 | 7.958 | 8.508 | 0.004 | 6.967 | 0.002 |
| 180 | 741.3673 | 11.703 | 14.129 | 0.025 | 16.408 | 0.000 |
| 181 | 741.3674 | 11.307 | 10.098 | 0.006 | 6.303 | 0.015 |
| 182 | 741.3674 | 11.863 | 33.069 | 0.001 | 19.658 | 0.004 |
| 183 | 743.3828 | 10.334 | 41.808 | 0.002 | 17.947 | 0.017 |
| 184 | 743.383 | 10.737 | 13.401 | 0.002 | 8.359 | 0.002 |
| 185 | 743.3831 | 10.343 | 37.954 | 0.002 | 17.215 | 0.015 |
| 186 | 758.3734 | 10.574 | 12.851 | 0.007 | 11.951 | 0.009 |
| 187 | 759.3779 | 9.417 | 48.094 | 0.000 | 18.728 | 0.015 |
| 188 | 759.378 | 9.416 | 48.897 | 0.000 | 19.041 | 0.015 |
| 189 | 759.3781 | 10.577 | 12.526 | 0.003 | 11.035 | 0.005 |
| 190 | 765.3343 | 10.896 | 29.876 | 0.008 | 7.135 | 0.020 |
| 191 | 780.2987 | 10.918 | 27.089 | 0.012 | 9.235 | 0.020 |
| 192 | 800.503 | 43.588 | 21.353 | 0.002 | 10.901 | 0.032 |
| 193 | 827.9458 | 1.03 | 5.834 | 0.004 | 5.926 | 0.044 |
| 194 | 863.9086 | 20.035 | 7.004 | 0.010 | 7.267 | 0.001 |
| 195 | 904.5368 | 33.639 | 10.715 | 0.003 | 8.698 | 0.001 |
| 196 | 1092.643 | 38.743 | 65.088 | 0.010 | 5.375 | 0.031 |

**Table S2.** Metabolites resulting from co-culture of *F. sporotichioides* 2006a and *Trichoderma* strains; green- metabolites specific to *Trichoderma*, pink- metabolites specific to *Fusarium*; F: *Fusarium* monoculture, T: *Trichoderma* monoculture, TF: co-culture; ↑/↓- increase/decrease in metabolite content in co-culture compared to control cultures

| # | **m/z** | **RT [min]** | **Ratio** | | | **P-value** | | | **Change in co-culture** |
| --- | --- | --- | --- | --- | --- | --- | --- | --- | --- |
|  |  |  | **(T)/(F)** | **(TF)/(F)** | **(TF)/(T)** | **(T)/(F)** | **(TF)/(F)** | **(TF)/(T)** |  |
| 1 | 111.0077 | 1.033 | 10.085 | 41.917 | 4.203 | 0.020 | 0.001 | 0.095 | ↑ |
| 2 | 121.1014 | 11.117 | 13.484 | 9.046 | 1.251 | 0.038 | 0.021 | 0.751 | - |
| 3 | 122.0601 | 1.316 | 14.123 | 98.495 | 4.306 | 0.029 | 0.021 | 0.026 | ↑ |
| 4 | 123.0636 | 1.301 | 17.888 | 127.238 | 4.361 | 0.038 | 0.025 | 0.027 | ↑ |
| 5 | 129.0184 | 1.036 | 11.113 | 20.312 | 4.485 | 0.037 | 0.010 | 0.089 | ↑ |
| 6 | 131.1293 | 0.692 | 57.027 | 79.444 | 1.594 | 0.005 | 0.001 | 0.121 | - |
| 7 | 138.0551 | 0.774 | 96.541 | 74.493 | 0.744 | 0.000 | 0.000 | 0.016 | - |
| 8 | 140.0707 | 1.294 | 14.199 | 92.851 | 4.076 | 0.028 | 0.021 | 0.025 | ↑ |
| 9 | 141.0741 | 1.295 | 33.957 | 142.028 | 4.183 | 0.034 | 0.047 | 0.088 | ↑ |
| 10 | 144.102 | 0.776 | 0.012 | 0.363 | 30.861 | 0.002 | 0.004 | 0.002 | ↓ |
| 11 | 146.1176 | 0.774 | 10.623 | 9.521 | 0.609 | 0.007 | 0.013 | 0.648 | - |
| 12 | 147.0289 | 1.037 | 10.761 | 23.494 | 4.585 | 0.028 | 0.007 | 0.094 | ↑ |
| 13 | 152.1071 | 1.918 | 10.283 | 15.808 | 2.317 | 0.016 | 0.007 | 0.127 | ↑ |
| 14 | 152.1072 | 1.609 | 15.494 | 26.892 | 1.598 | 0.017 | 0.014 | 0.080 | - |
| 15 | 157.0133 | 1.037 | 17.575 | 38.406 | 4.168 | 0.043 | 0.009 | 0.080 | ↑ |
| 16 | 158.1177 | 0.771 | 0.036 | 0.294 | 13.296 | 0.002 | 0.060 | 0.010 | ↓ |
| 17 | 158.1178 | 1.039 | 0.068 | 0.370 | 5.382 | 0.006 | 0.142 | 0.079 | ↓ |
| 18 | 163.0756 | 14.839 | 0.013 | 0.122 | 10.415 | 0.045 | 0.433 | 0.028 | ↓ |
| 19 | 166.084 | 0.753 | 0.010 | 0.208 | 21.240 | 0.001 | 0.002 | 0.001 | ↓ |
| 20 | 166.1228 | 2.162 | 60.169 | 107.639 | 1.789 | 0.006 | 0.024 | 0.471 | - |
| 21 | 166.1228 | 1.739 | 41.908 | 102.703 | 2.371 | 0.008 | 0.019 | 0.155 | ↑ |
| 22 | 171.0631 | 1.143 | 0.042 | 0.354 | 8.326 | 0.039 | 0.171 | 0.007 | ↓ |
| 23 | 171.1244 | 1.132 | 35.694 | 28.668 | 0.473 | 0.011 | 0.044 | 0.256 | ↓ |
| 24 | 181.086 | 15.726 | 0.088 | 0.303 | 4.592 | 0.009 | 0.102 | 0.015 | ↓ |
| 25 | 193.0344 | 0.796 | 14.250 | 26.769 | 4.239 | 0.013 | 0.010 | 0.112 | ↑ |
| 26 | 194.1656 | 2.527 | 0.050 | 0.056 | 1.052 | 0.001 | 0.001 | 0.073 | ↓ |
| 27 | 195.0656 | 12.803 | 0.026 | 0.022 | 0.849 | 0.027 | 0.022 | 0.018 | ↓ |
| 28 | 195.1746 | 16.634 | 14.278 | 80.901 | 4.557 | 0.017 | 0.000 | 0.034 | ↑ |
| 29 | 197.081 | 10.739 | 17.822 | 111.461 | 2.251 | 0.021 | 0.031 | 0.195 | ↑ |
| 30 | 198.1603 | 2.045 | 27.390 | 77.226 | 3.211 | 0.005 | 0.010 | 0.040 | ↑ |
| 31 | 204.0869 | 16.785 | 12.711 | 17.038 | 1.070 | 0.002 | 0.007 | 0.662 | - |
| 32 | 204.1231 | 0.782 | 24.600 | 19.145 | 0.778 | 0.004 | 0.010 | 0.240 | - |
| 33 | 204.1234 | 1.055 | 19.177 | 23.968 | 1.261 | 0.001 | 0.000 | 0.140 | - |
| 34 | 205.1187 | 1.026 | 0.044 | 0.025 | 1.425 | 0.019 | 0.015 | 0.784 | ↓ |
| 35 | 206.139 | 1.085 | 11.815 | 19.884 | 1.478 | 0.000 | 0.010 | 0.398 | - |
| 36 | 207.1861 | 16.798 | 0.016 | 0.016 | 0.988 | 0.016 | 0.016 | 0.942 | ↓ |
| 37 | 207.1861 | 17.783 | 0.034 | 0.035 | 1.004 | 0.025 | 0.023 | 0.977 | ↓ |
| 38 | 208.0971 | 12.21 | 20.702 | 25.836 | 1.073 | 0.003 | 0.001 | 0.715 | - |
| 39 | 213.1853 | 16.544 | 17.073 | 59.459 | 2.444 | 0.017 | 0.026 | 0.334 | ↑ |
| 40 | 215.0166 | 0.817 | 20.461 | 37.077 | 3.629 | 0.028 | 0.005 | 0.076 | ↑ |
| 41 | 215.1393 | 5.813 | 10.384 | 21.483 | 2.146 | 0.020 | 0.012 | 0.008 | ↑ |
| 42 | 218.139 | 1.644 | 11.095 | 13.291 | 1.382 | 0.001 | 0.005 | 0.146 | - |
| 43 | 220.1196 | 7.02 | 26.863 | 28.545 | 1.553 | 0.023 | 0.048 | 0.506 | - |
| 44 | 221.1902 | 11.281 | 14.124 | 51.525 | 2.951 | 0.002 | 0.001 | 0.006 | ↑ |
| 45 | 223.2062 | 17.272 | 0.012 | 0.116 | 6.985 | 0.043 | 0.159 | 0.002 | ↓ |
| 46 | 224.0781 | 5.954 | 124.196 | 131.852 | 1.147 | 0.026 | 0.006 | 0.343 | - |
| 47 | 224.0921 | 7.464 | 17.019 | 30.577 | 2.236 | 0.001 | 0.002 | 0.048 | ↑ |
| 48 | 226.1189 | 8.236 | 40.946 | 56.092 | 1.370 | 0.003 | 0.000 | 0.087 | - |
| 49 | 226.1189 | 7.789 | 22.508 | 39.913 | 1.773 | 0.007 | 0.001 | 0.051 | - |
| 50 | 226.1442 | 2.942 | 18.320 | 15.864 | 1.238 | 0.011 | 0.002 | 0.430 | - |
| 51 | 227.0504 | 0.713 | 34.933 | 38.863 | 1.250 | 0.013 | 0.007 | 0.719 | - |
| 52 | 231.1343 | 5.779 | 21.909 | 30.715 | 1.783 | 0.015 | 0.014 | 0.047 | - |
| 53 | 232.0609 | 11.536 | 0.042 | 0.044 | 1.033 | 0.011 | 0.010 | 0.269 | ↓ |
| 54 | 232.1183 | 7.065 | 23.231 | 45.308 | 1.988 | 0.006 | 0.003 | 0.012 | - |
| 55 | 232.1545 | 5.664 | 18.194 | 11.019 | 1.319 | 0.034 | 0.023 | 0.819 | - |
| 56 | 233.0448 | 13.13 | 0.056 | 0.058 | 1.041 | 0.002 | 0.012 | 0.374 | ↓ |
| 57 | 235.0968 | 13.605 | 0.075 | 0.249 | 3.208 | 0.005 | 0.036 | 0.018 | ↓ |
| 58 | 236.1494 | 0.782 | 41.017 | 31.838 | 0.674 | 0.027 | 0.038 | 0.025 | - |
| 59 | 236.2126 | 12.773 | 0.033 | 0.057 | 1.656 | 0.010 | 0.022 | 0.125 | ↓ |
| 60 | 237.1601 | 18.513 | 0.042 | 0.162 | 3.162 | 0.027 | 0.057 | 0.008 | ↓ |
| 61 | 237.1602 | 15.774 | 0.060 | 0.151 | 2.420 | 0.038 | 0.081 | 0.013 | ↓ |
| 62 | 237.1603 | 18.507 | 0.037 | 0.134 | 3.098 | 0.030 | 0.069 | 0.003 | ↓ |
| 63 | 237.1853 | 12.637 | 0.023 | 0.118 | 4.493 | 0.036 | 0.110 | 0.001 | ↓ |
| 64 | 239.1758 | 15.462 | 0.063 | 0.137 | 2.881 | 0.024 | 0.032 | 0.018 | ↓ |
| 65 | 243.1706 | 7.957 | 13.359 | 78.488 | 5.875 | 0.040 | 0.023 | 0.007 | ↑ |
| 66 | 245.1176 | 11.642 | 0.022 | 0.057 | 2.088 | 0.020 | 0.026 | 0.025 | ↓ |
| 67 | 246.1606 | 13.495 | 0.031 | 0.114 | 3.751 | 0.000 | 0.008 | 0.032 | ↓ |
| 68 | 247.2019 | 0.755 | 0.004 | 0.119 | 33.232 | 0.000 | 0.004 | 0.003 | ↓ |
| 69 | 248.1495 | 1.4 | 19.950 | 11.764 | 0.590 | 0.017 | 0.013 | 0.570 | - |
| 70 | 255.1958 | 21.692 | 18.013 | 34.120 | 1.697 | 0.001 | 0.000 | 0.048 | - |
| 71 | 257.1864 | 15.292 | 0.042 | 0.116 | 2.779 | 0.023 | 0.025 | 0.019 | ↓ |
| 72 | 259.1293 | 6.669 | 36.839 | 21.736 | 0.753 | 0.001 | 0.003 | 0.199 | - |
| 73 | 260.1132 | 5.531 | 41.248 | 45.120 | 1.094 | 0.004 | 0.009 | 0.555 | - |
| 74 | 261.1448 | 2.002 | 44.861 | 84.929 | 2.230 | 0.026 | 0.016 | 0.043 | ↑ |
| 75 | 261.1448 | 1.708 | 41.467 | 112.297 | 1.738 | 0.034 | 0.026 | 0.046 | - |
| 76 | 261.1812 | 0.756 | 0.066 | 1.276 | 17.230 | 0.033 | 0.217 | 0.010 | - |
| 77 | 262.1653 | 2.384 | 12.244 | 15.110 | 1.124 | 0.000 | 0.006 | 0.330 | - |
| 78 | 263.1392 | 10.545 | 17.982 | 4.447 | 0.187 | 0.010 | 0.140 | 0.060 | ↓ |
| 79 | 263.201 | 27.83 | 0.073 | 0.122 | 1.642 | 0.003 | 0.007 | 0.028 | ↓ |
| 80 | 264.1964 | 14.399 | 0.054 | 0.129 | 2.176 | 0.003 | 0.011 | 0.020 | ↓ |
| 81 | 265.1916 | 14.532 | 0.015 | 0.016 | 0.931 | 0.005 | 0.003 | 0.818 | ↓ |
| 82 | 265.2028 | 17.618 | 0.067 | 0.092 | 1.166 | 0.037 | 0.039 | 0.628 | ↓ |
| 83 | 271.1294 | 1.032 | 0.023 | 0.059 | 4.608 | 0.020 | 0.139 | 0.071 | ↓ |
| 84 | 273.1925 | 0.771 | 0.016 | 0.313 | 17.999 | 0.009 | 0.122 | 0.000 | ↓ |
| 85 | 277.0896 | 0.752 | 13.709 | 2.544 | 0.186 | 0.001 | 0.039 | 0.003 | ↓ |
| 86 | 277.1527 | 15.776 | 0.071 | 0.159 | 2.284 | 0.042 | 0.052 | 0.022 | ↓ |
| 87 | 282.1706 | 13.925 | 0.017 | 0.019 | 1.234 | 0.023 | 0.029 | 0.140 | ↓ |
| 88 | 284.1974 | 12.688 | 0.037 | 0.038 | 1.012 | 0.005 | 0.004 | 0.743 | ↓ |
| 89 | 285.2291 | 14.309 | 0.087 | 0.091 | 1.158 | 0.010 | 0.009 | 0.583 | ↓ |
| 90 | 286.1435 | 0.721 | 0.090 | 0.199 | 1.711 | 0.016 | 0.004 | 0.187 | ↓ |
| 91 | 287.0557 | 16.972 | 0.016 | 0.017 | 1.066 | 0.014 | 0.013 | 0.090 | ↓ |
| 92 | 289.0974 | 10.596 | 16.235 | 2.699 | 0.166 | 0.000 | 0.072 | 0.029 | ↓ |
| 93 | 290.212 | 21.592 | 0.058 | 0.085 | 1.468 | 0.011 | 0.027 | 0.263 | ↓ |
| 94 | 290.233 | 12.635 | 0.036 | 0.089 | 2.476 | 0.037 | 0.104 | 0.008 | ↓ |
| 95 | 293.1727 | 16.916 | 0.079 | 0.474 | 3.821 | 0.024 | 0.254 | 0.227 | ↓ |
| 96 | 295.1425 | 15.464 | 0.032 | 0.114 | 3.273 | 0.006 | 0.022 | 0.007 | ↓ |
| 97 | 295.1884 | 12.625 | 0.012 | 0.090 | 9.607 | 0.017 | 0.105 | 0.004 | ↓ |
| 98 | 298.2744 | 38.696 | 0.066 | 0.065 | 0.995 | 0.003 | 0.008 | 0.664 | ↓ |
| 99 | 301.2128 | 15.497 | 0.033 | 0.034 | 0.990 | 0.003 | 0.003 | 0.986 | ↓ |
| 100 | 302.2077 | 10.312 | 13.391 | 10.466 | 0.782 | 0.041 | 0.007 | 0.723 | - |
| 101 | 305.2074 | 0.763 | 0.086 | 1.795 | 21.726 | 0.004 | 0.004 | 0.002 | ↓ |
| 102 | 308.2224 | 20.013 | 0.021 | 0.030 | 1.324 | 0.021 | 0.017 | 0.349 | ↓ |
| 103 | 308.2226 | 34.976 | 0.033 | 0.034 | 1.301 | 0.024 | 0.032 | 0.211 | ↓ |
| 104 | 308.2227 | 20.599 | 0.060 | 0.054 | 0.590 | 0.008 | 0.015 | 0.184 | ↓ |
| 105 | 308.2227 | 19.732 | 0.028 | 0.051 | 0.743 | 0.010 | 0.013 | 0.994 | ↓ |
| 106 | 309.1816 | 14.068 | 0.066 | 0.099 | 1.049 | 0.039 | 0.046 | 0.354 | ↓ |
| 107 | 312.1812 | 17.7 | 0.048 | 0.160 | 2.298 | 0.022 | 0.061 | 0.066 | ↓ |
| 108 | 317.2077 | 9.961 | 0.041 | 0.048 | 1.180 | 0.007 | 0.010 | 0.056 | ↓ |
| 109 | 317.2078 | 11.792 | 0.037 | 0.038 | 0.992 | 0.008 | 0.011 | 0.475 | ↓ |
| 110 | 320.1609 | 6.675 | 10.124 | 24.786 | 2.226 | 0.002 | 0.002 | 0.054 | ↑ |
| 111 | 322.1764 | 8.183 | 107.124 | 128.039 | 1.578 | 0.030 | 0.014 | 0.161 | - |
| 112 | 322.1765 | 7.871 | 104.775 | 176.964 | 1.689 | 0.037 | 0.017 | 0.173 | - |
| 113 | 323.1948 | 15.593 | 0.013 | 0.013 | 0.999 | 0.001 | 0.001 | 0.444 | ↓ |
| 114 | 324.2176 | 18.232 | 0.091 | 0.074 | 0.653 | 0.024 | 0.029 | 0.193 | ↓ |
| 115 | 327.159 | 0.755 | 0.028 | 0.181 | 6.433 | 0.001 | 0.012 | 0.001 | ↓ |
| 116 | 336.1558 | 7.402 | 21.011 | 81.442 | 3.236 | 0.005 | 0.012 | 0.047 | ↑ |
| 117 | 336.8521 | 0.715 | 10.360 | 9.177 | 0.708 | 0.008 | 0.007 | 0.145 | - |
| 118 | 338.1715 | 7.788 | 111.562 | 154.612 | 1.386 | 0.030 | 0.016 | 0.134 | - |
| 119 | 338.1717 | 8.229 | 132.184 | 172.331 | 1.304 | 0.028 | 0.015 | 0.146 | - |
| 120 | 338.3061 | 43.209 | 0.033 | 0.088 | 3.355 | 0.011 | 0.029 | 0.014 | ↓ |
| 121 | 339.3013 | 38.498 | 0.034 | 0.053 | 1.899 | 0.008 | 0.004 | 0.054 | ↓ |
| 122 | 340.3216 | 46.512 | 0.048 | 0.085 | 1.783 | 0.002 | 0.011 | 0.026 | ↓ |
| 123 | 341.3167 | 42.133 | 0.089 | 0.226 | 3.048 | 0.008 | 0.014 | 0.008 | ↓ |
| 124 | 342.2391 | 12.517 | 12.219 | 14.644 | 1.080 | 0.005 | 0.001 | 0.974 | - |
| 125 | 352.1329 | 5.148 | 10.294 | 18.508 | 1.642 | 0.041 | 0.000 | 0.366 | - |
| 126 | 352.133 | 7.309 | 16.409 | 40.715 | 2.382 | 0.001 | 0.000 | 0.001 | ↑ |
| 127 | 353.2285 | 0.763 | 27.371 | 20.213 | 0.730 | 0.000 | 0.001 | 0.003 | - |
| 128 | 353.2443 | 17.497 | 0.042 | 0.043 | 1.005 | 0.028 | 0.028 | 0.940 | ↓ |
| 129 | 356.2184 | 7.956 | 13.107 | 109.059 | 3.994 | 0.014 | 0.003 | 0.038 | ↑ |
| 130 | 357.2024 | 9.238 | 22.170 | 103.894 | 4.006 | 0.012 | 0.001 | 0.030 | ↑ |
| 131 | 362.2333 | 15.929 | 0.069 | 0.070 | 1.012 | 0.005 | 0.005 | 0.639 | ↓ |
| 132 | 362.2334 | 16.067 | 0.073 | 0.074 | 0.952 | 0.008 | 0.007 | 0.289 | ↓ |
| 133 | 363.1725 | 8.186 | 26.477 | 37.975 | 1.393 | 0.014 | 0.013 | 0.637 | - |
| 134 | 363.1726 | 7.875 | 13.449 | 24.856 | 1.628 | 0.013 | 0.025 | 0.595 | - |
| 135 | 363.3013 | 37.651 | 0.042 | 0.117 | 2.512 | 0.004 | 0.006 | 0.015 | ↓ |
| 136 | 365.3168 | 40.366 | 0.083 | 0.235 | 3.199 | 0.002 | 0.003 | 0.016 | ↓ |
| 137 | 366.1854 | 17.99 | 0.095 | 0.143 | 2.688 | 0.012 | 0.093 | 0.109 | ↓ |
| 138 | 367.3325 | 43.561 | 0.038 | 0.183 | 4.533 | 0.002 | 0.009 | 0.000 | ↓ |
| 139 | 368.2185 | 8.297 | 11.781 | 10.270 | 0.783 | 0.024 | 0.024 | 0.092 | - |
| 140 | 368.336 | 43.544 | 0.038 | 0.182 | 5.266 | 0.004 | 0.007 | 0.002 | ↓ |
| 141 | 369.1596 | 1.127 | 12.413 | 19.532 | 1.129 | 0.024 | 0.015 | 0.770 | - |
| 142 | 371.1929 | 8.826 | 19.961 | 19.829 | 0.766 | 0.007 | 0.007 | 0.725 | - |
| 143 | 372.1899 | 9.157 | 20.107 | 233.508 | 13.489 | 0.015 | 0.007 | 0.031 | ↑ |
| 144 | 372.2753 | 27.829 | 0.061 | 0.164 | 2.684 | 0.015 | 0.011 | 0.040 | ↓ |
| 145 | 373.1973 | 9.155 | 30.452 | 414.524 | 15.310 | 0.009 | 0.000 | 0.033 | ↑ |
| 146 | 374.218 | 11.56 | 0.048 | 0.066 | 1.361 | 0.024 | 0.037 | 0.044 | ↓ |
| 147 | 374.2911 | 23.814 | 0.082 | 0.122 | 1.536 | 0.001 | 0.001 | 0.039 | ↓ |
| 148 | 376.2489 | 17.841 | 0.011 | 0.055 | 3.872 | 0.004 | 0.008 | 0.004 | ↓ |
| 149 | 377.2806 | 18.363 | 0.022 | 0.029 | 1.076 | 0.009 | 0.015 | 0.426 | ↓ |
| 150 | 379.116 | 9.36 | 0.016 | 0.015 | 1.266 | 0.035 | 0.076 | 0.351 | ↓ |
| 151 | 380.3313 | 41.352 | 10.315 | 11.883 | 1.152 | 0.031 | 0.006 | 0.816 | - |
| 152 | 385.2087 | 6.929 | 13.997 | 16.608 | 1.187 | 0.000 | 0.004 | 0.523 | - |
| 153 | 385.343 | 40.316 | 0.042 | 0.093 | 2.205 | 0.008 | 0.070 | 0.096 | ↓ |
| 154 | 393.1522 | 10.94 | 15.126 | 27.355 | 1.495 | 0.005 | 0.002 | 0.065 | - |
| 155 | 396.2498 | 8.051 | 10.785 | 8.274 | 0.767 | 0.019 | 0.002 | 0.354 | - |
| 156 | 397.1544 | 1.044 | 19.546 | 47.040 | 1.664 | 0.018 | 0.013 | 0.209 | - |
| 157 | 397.6193 | 7.969 | 25.851 | 63.129 | 2.696 | 0.003 | 0.000 | 0.040 | ↑ |
| 158 | 398.2025 | 1.022 | 17.974 | 40.979 | 2.243 | 0.005 | 0.000 | 0.072 | ↑ |
| 159 | 398.229 | 10.683 | 30.120 | 34.638 | 1.412 | 0.037 | 0.012 | 0.177 | - |
| 160 | 398.2907 | 18.408 | 0.068 | 0.216 | 3.511 | 0.003 | 0.004 | 0.007 | ↓ |
| 161 | 399.1628 | 9.689 | 28.916 | 28.644 | 1.257 | 0.001 | 0.004 | 0.260 | - |
| 162 | 399.213 | 12.328 | 15.411 | 15.644 | 0.907 | 0.000 | 0.017 | 0.431 | - |
| 163 | 399.2244 | 7.551 | 39.772 | 46.814 | 1.177 | 0.004 | 0.006 | 0.115 | - |
| 164 | 400.1971 | 11.613 | 0.004 | 0.145 | 30.448 | 0.027 | 0.130 | 0.000 | ↓ |
| 165 | 400.1971 | 12.309 | 0.002 | 0.019 | 9.847 | 0.011 | 0.046 | 0.005 | ↓ |
| 166 | 400.1973 | 13.728 | 0.016 | 0.016 | 0.999 | 0.026 | 0.026 | 0.772 | ↓ |
| 167 | 400.1973 | 12.534 | 0.014 | 0.016 | 1.111 | 0.024 | 0.031 | 0.136 | ↓ |
| 168 | 405.1527 | 12.528 | 0.009 | 0.010 | 1.142 | 0.018 | 0.034 | 0.249 | ↓ |
| 169 | 408.3228 | 22.023 | 0.044 | 0.046 | 1.051 | 0.002 | 0.001 | 0.429 | ↓ |
| 170 | 409.343 | 38.616 | 0.020 | 0.043 | 3.922 | 0.005 | 0.009 | 0.033 | ↓ |
| 171 | 409.3434 | 39.475 | 0.062 | 0.081 | 1.302 | 0.002 | 0.010 | 0.178 | ↓ |
| 172 | 410.375 | 40.371 | 0.052 | 0.083 | 1.551 | 0.000 | 0.009 | 0.250 | ↓ |
| 173 | 411.3585 | 41.451 | 0.019 | 0.050 | 2.064 | 0.004 | 0.011 | 0.028 | ↓ |
| 174 | 411.359 | 42.632 | 0.045 | 0.051 | 1.063 | 0.000 | 0.001 | 0.209 | ↓ |
| 175 | 412.3906 | 43.555 | 0.029 | 0.058 | 2.578 | 0.003 | 0.010 | 0.019 | ↓ |
| 176 | 413.288 | 43.2 | 11.956 | 17.258 | 0.989 | 0.023 | 0.026 | 0.511 | - |
| 177 | 415.3061 | 41.272 | 12.055 | 15.701 | 1.111 | 0.032 | 0.019 | 0.976 | - |
| 178 | 421.1265 | 12.308 | 0.004 | 0.021 | 4.758 | 0.013 | 0.047 | 0.020 | ↓ |
| 179 | 421.1266 | 13.963 | 0.006 | 0.007 | 0.958 | 0.036 | 0.035 | 0.983 | ↓ |
| 180 | 421.1268 | 12.529 | 0.023 | 0.033 | 1.114 | 0.035 | 0.035 | 0.241 | ↓ |
| 181 | 423.0086 | 1.037 | 35.933 | 154.314 | 7.985 | 0.031 | 0.003 | 0.091 | ↑ |
| 182 | 428.2285 | 12.345 | 0.002 | 0.020 | 8.215 | 0.012 | 0.046 | 0.014 | ↓ |
| 183 | 428.2287 | 12.531 | 0.014 | 0.016 | 1.098 | 0.024 | 0.023 | 0.147 | ↓ |
| 184 | 431.3121 | 19.999 | 0.045 | 0.168 | 3.779 | 0.019 | 0.061 | 0.006 | ↓ |
| 185 | 432.3325 | 41.275 | 13.691 | 16.521 | 1.066 | 0.001 | 0.007 | 0.946 | - |
| 186 | 439.3325 | 35.635 | 55.984 | 73.340 | 1.364 | 0.002 | 0.002 | 0.130 | - |
| 187 | 439.3326 | 33.776 | 0.037 | 0.486 | 9.334 | 0.015 | 0.112 | 0.006 | ↓ |
| 188 | 440.251 | 11.245 | 13.755 | 11.022 | 0.765 | 0.008 | 0.000 | 0.309 | - |
| 189 | 440.3357 | 35.638 | 22.213 | 36.494 | 1.363 | 0.003 | 0.002 | 0.114 | - |
| 190 | 451.2557 | 9.554 | 19.655 | 40.072 | 2.039 | 0.002 | 0.000 | 0.005 | ↑ |
| 191 | 453.2712 | 9.778 | 419.199 | 557.201 | 1.321 | 0.044 | 0.038 | 0.190 | - |
| 192 | 460.3639 | 38.225 | 10.573 | 9.663 | 0.756 | 0.013 | 0.021 | 0.402 | - |
| 193 | 467.2506 | 10.633 | 11.529 | 23.669 | 2.009 | 0.002 | 0.000 | 0.057 | ↑ |
| 194 | 469.266 | 9.697 | 218.972 | 172.101 | 0.846 | 0.020 | 0.024 | 0.773 | - |
| 195 | 479.2776 | 45.981 | 0.047 | 0.060 | 1.031 | 0.001 | 0.004 | 0.864 | ↓ |
| 196 | 481.2299 | 10.583 | 31.163 | 125.811 | 2.509 | 0.003 | 0.002 | 0.048 | ↑ |
| 197 | 481.23 | 9.659 | 25.585 | 92.282 | 2.055 | 0.014 | 0.007 | 0.118 | ↑ |
| 198 | 481.23 | 7.484 | 10.642 | 27.159 | 1.935 | 0.026 | 0.003 | 0.151 | - |
| 199 | 481.3647 | 25.35 | 0.020 | 0.020 | 1.006 | 0.022 | 0.021 | 0.803 | ↓ |
| 200 | 481.3647 | 25.714 | 0.026 | 0.026 | 1.010 | 0.024 | 0.025 | 0.793 | ↓ |
| 201 | 483.2453 | 8.832 | 19.976 | 45.514 | 2.278 | 0.001 | 0.004 | 0.124 | ↑ |
| 202 | 483.2454 | 10.61 | 28.615 | 65.443 | 1.442 | 0.032 | 0.031 | 0.185 | - |
| 203 | 484.2536 | 9.642 | 15.323 | 51.613 | 1.776 | 0.022 | 0.006 | 0.260 | - |
| 204 | 485.2606 | 9.635 | 69.598 | 90.523 | 1.424 | 0.001 | 0.010 | 0.281 | - |
| 205 | 498.7616 | 41.269 | 15.697 | 14.429 | 1.110 | 0.002 | 0.001 | 0.795 | - |
| 206 | 499.2405 | 10.078 | 12.922 | 65.378 | 3.346 | 0.006 | 0.003 | 0.039 | ↑ |
| 207 | 499.2588 | 41.269 | 14.010 | 14.721 | 1.087 | 0.002 | 0.001 | 0.856 | - |
| 208 | 501.2561 | 8.841 | 14.176 | 28.438 | 1.297 | 0.018 | 0.004 | 0.295 | - |
| 209 | 501.2562 | 9.411 | 15.263 | 44.306 | 2.715 | 0.009 | 0.003 | 0.039 | ↑ |
| 210 | 501.2562 | 10.622 | 19.725 | 13.648 | 0.692 | 0.003 | 0.005 | 0.001 | - |
| 211 | 506.3249 | 34.408 | 46.173 | 53.639 | 1.162 | 0.000 | 0.001 | 0.758 | - |
| 212 | 506.8768 | 9.706 | 18.849 | 21.795 | 0.961 | 0.001 | 0.002 | 0.584 | - |
| 213 | 507.2129 | 9.701 | 22.300 | 25.034 | 1.001 | 0.001 | 0.002 | 0.635 | - |
| 214 | 508.3407 | 37.032 | 17.622 | 15.791 | 0.951 | 0.001 | 0.002 | 0.493 | - |
| 215 | 514.3745 | 30.804 | 12.996 | 16.468 | 1.128 | 0.009 | 0.014 | 0.219 | - |
| 216 | 518.3249 | 28.683 | 10.612 | 7.802 | 0.902 | 0.009 | 0.015 | 0.525 | - |
| 217 | 520.976 | 1.035 | 10.307 | 85.278 | 8.310 | 0.027 | 0.003 | 0.158 | ↑ |
| 218 | 523.2076 | 9.636 | 115.297 | 314.211 | 1.528 | 0.004 | 0.002 | 0.225 | - |
| 219 | 523.2078 | 9.635 | 105.924 | 284.099 | 1.489 | 0.005 | 0.002 | 0.245 | - |
| 220 | 531.2212 | 32.497 | 0.003 | 0.003 | 1.064 | 0.008 | 0.008 | 0.106 | ↓ |
| 221 | 532.188 | 1.342 | 65.171 | 7.776 | 0.137 | 0.001 | 0.017 | 0.038 | ↓ |
| 222 | 538.1725 | 9.643 | 71.017 | 127.186 | 1.174 | 0.003 | 0.002 | 0.304 | - |
| 223 | 542.3199 | 36.472 | 160.876 | 374.236 | 3.888 | 0.034 | 0.002 | 0.300 | ↑ |
| 224 | 555.0931 | 27.117 | 0.014 | 0.014 | 1.035 | 0.016 | 0.015 | 0.122 | ↓ |
| 225 | 555.3038 | 13.19 | 0.038 | 0.092 | 1.477 | 0.018 | 0.024 | 0.090 | ↓ |
| 226 | 557.0725 | 20.682 | 0.002 | 0.004 | 1.102 | 0.002 | 0.002 | 0.316 | ↓ |
| 227 | 557.1084 | 34.164 | 0.052 | 0.063 | 1.237 | 0.017 | 0.022 | 0.021 | ↓ |
| 228 | 571.0878 | 29.133 | 0.001 | 0.050 | 56.644 | 0.013 | 0.145 | 0.004 | ↓ |
| 229 | 587.083 | 23.63 | 0.034 | 0.035 | 1.020 | 0.026 | 0.026 | 0.054 | ↓ |
| 230 | 591.1957 | 9.634 | 13.719 | 38.154 | 1.721 | 0.006 | 0.003 | 0.109 | - |
| 231 | 598.3091 | 10.074 | 16.156 | 160.301 | 7.364 | 0.002 | 0.000 | 0.003 | ↑ |
| 232 | 604.2975 | 20.433 | 0.019 | 0.058 | 4.181 | 0.038 | 0.137 | 0.030 | ↓ |
| 233 | 607.1607 | 9.634 | 46.091 | 105.521 | 1.427 | 0.005 | 0.002 | 0.226 | - |
| 234 | 632.2926 | 22.446 | 0.028 | 0.026 | 0.965 | 0.007 | 0.008 | 0.210 | ↓ |
| 235 | 644.4742 | 45.392 | 13.817 | 11.763 | 0.869 | 0.040 | 0.049 | 0.162 | - |
| 236 | 647.3142 | 9.296 | 55.975 | 61.828 | 1.105 | 0.001 | 0.004 | 0.312 | - |
| 237 | 679.403 | 11.078 | 20.554 | 109.611 | 3.616 | 0.026 | 0.003 | 0.105 | ↑ |
| 238 | 691.2973 | 23.918 | 0.032 | 0.032 | 1.001 | 0.023 | 0.023 | 0.651 | ↓ |
| 239 | 695.3979 | 11.022 | 22.323 | 252.383 | 8.895 | 0.002 | 0.003 | 0.018 | ↑ |
| 240 | 702.3424 | 8.798 | 26.924 | 81.835 | 3.830 | 0.010 | 0.000 | 0.052 | ↑ |
| 241 | 702.3426 | 7.513 | 40.350 | 54.885 | 1.357 | 0.003 | 0.000 | 0.248 |  |
| 242 | 711.3927 | 10.967 | 40.960 | 654.530 | 15.980 | 0.013 | 0.000 | 0.014 | ↑ |
| 243 | 718.3371 | 7.962 | 76.436 | 243.987 | 2.640 | 0.011 | 0.010 | 0.029 | ↑ |
| 244 | 727.3868 | 10.903 | 80.018 | 923.432 | 8.931 | 0.018 | 0.007 | 0.032 | ↑ |
| 245 | 756.2846 | 7.968 | 31.637 | 80.629 | 2.845 | 0.004 | 0.000 | 0.036 | ↑ |
| 246 | 775.4352 | 39.561 | 28.440 | 7.650 | 0.269 | 0.039 | 0.349 | 0.076 | ↓ |
| 247 | 788.4673 | 39.871 | 13.162 | 64.260 | 3.000 | 0.038 | 0.007 | 0.142 | ↑ |
| 248 | 789.4512 | 40.478 | 16.823 | 6.582 | 0.478 | 0.004 | 0.172 | 0.257 | ↓ |
| 249 | 789.4514 | 42.308 | 15.772 | 4.741 | 0.462 | 0.003 | 0.025 | 0.087 | ↓ |
| 250 | 816.499 | 39.552 | 21.158 | 2.089 | 0.129 | 0.038 | 0.473 | 0.002 | ↓ |
| 251 | 841.4022 | 37.109 | 0.033 | 0.040 | 1.387 | 0.015 | 0.018 | 0.031 | ↓ |
| 252 | 841.4942 | 40.519 | 14.349 | 1.642 | 0.114 | 0.016 | 0.146 | 0.005 | ↓ |
| 253 | 874.5301 | 38.089 | 0.021 | 0.030 | 1.262 | 0.005 | 0.008 | 0.161 | ↓ |
| 254 | 876.4396 | 37.112 | 0.076 | 0.102 | 1.345 | 0.038 | 0.050 | 0.026 | ↓ |
| 255 | 888.5455 | 39.378 | 0.002 | 0.008 | 3.313 | 0.006 | 0.008 | 0.006 | ↓ |
| 256 | 888.5455 | 39.615 | 0.003 | 0.010 | 3.441 | 0.002 | 0.000 | 0.051 | ↓ |
| 257 | 941.0483 | 40.317 | 14.305 | 4.491 | 0.314 | 0.031 | 0.228 | 0.064 | ↓ |
| 258 | 955.0734 | 40.424 | 10.241 | 2.134 | 0.202 | 0.015 | 0.542 | 0.029 | ↓ |
| 259 | 956.0528 | 41.414 | 12.664 | 3.662 | 0.663 | 0.017 | 0.143 | 0.440 | - |
| 260 | 963.0606 | 41.414 | 14.531 | 13.364 | 0.920 | 0.005 | 0.018 | 0.798 | - |
| 261 | 963.5622 | 42.483 | 11.413 | 11.498 | 0.993 | 0.014 | 0.040 | 0.423 | - |
| 262 | 969.5581 | 39.561 | 31.595 | 4.339 | 0.137 | 0.010 | 0.202 | 0.048 | ↓ |
| 263 | 970.0553 | 36.93 | 11.954 | 5.118 | 0.364 | 0.035 | 0.008 | 0.468 | ↓ |
| 264 | 971.5725 | 38.968 | 13.352 | 11.135 | 0.737 | 0.014 | 0.022 | 0.689 | - |
| 265 | 975.5715 | 40.759 | 25.198 | 48.300 | 1.360 | 0.023 | 0.019 | 0.211 | - |
| 266 | 982.0741 | 40.511 | 14.067 | 1.571 | 0.105 | 0.015 | 0.066 | 0.012 | ↓ |
| 267 | 983.0836 | 42.294 | 10.149 | 3.741 | 0.329 | 0.011 | 0.676 | 0.135 | ↓ |
| 268 | 984.5871 | 39.873 | 21.458 | 16.061 | 1.490 | 0.031 | 0.008 | 0.737 | - |
| 269 | 992.0873 | 42.309 | 14.956 | 2.109 | 0.141 | 0.031 | 0.783 | 0.069 | ↓ |
| 270 | 1092.643 | 39.479 | 23.767 | 12.322 | 0.518 | 0.018 | 0.048 | 0.100 | - |
| 271 | 1092.643 | 40.321 | 17.158 | 9.121 | 0.479 | 0.024 | 0.184 | 0.364 | ↓ |
| 272 | 1094.622 | 40.426 | 11.493 | 1.964 | 0.106 | 0.011 | 0.181 | 0.016 | ↓ |
| 273 | 1108.638 | 40.448 | 13.845 | 1.901 | 0.187 | 0.042 | 0.344 | 0.045 | ↓ |
| 274 | 1122.654 | 40.513 | 11.362 | 1.704 | 0.150 | 0.017 | 0.017 | 0.018 | ↓ |
| 275 | 1163.679 | 39.866 | 11.835 | 6.417 | 0.683 | 0.025 | 0.008 | 0.277 | - |


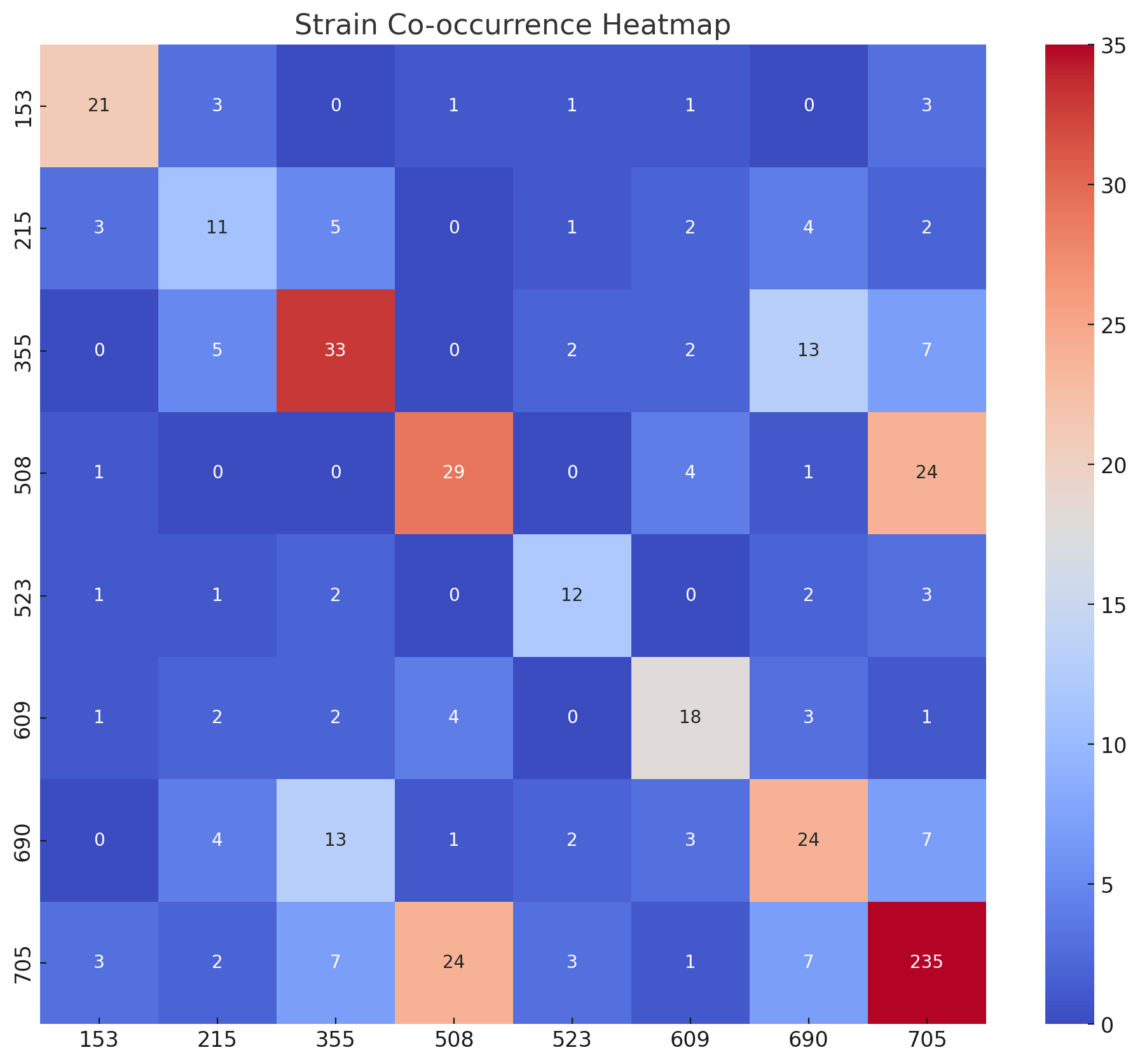


**Figure S1.** Heatmap showing the amount of common metabolites formed by the application of the antagonist in co-culture with the pathogen in metabolomic profiles AN153- *T. atroviride* AN153, AN215- *T. atroviride* AN215, AN355- *T. viride* AN355, AN508- *T. viridescens* AN508, AN523- *T*. *atroviride* AN523, AN609*- T. viridescens* AN609, AN690- *T. viride* 690, AN705- *T*. *atroviride* AN705.

**Table S3.** The list of characteristic metabolites formed in co-cultures.

| # | m/z | RT [min] |
| --- | --- | --- |
| 1 | 489.1764 | 27.046 |
| 2 | 489.1760 | 26.892 |
| 3 | 475.1605 | 24.218 |
| 4 | 511.1583 | 29.121 |
| 5 | 511.1584 | 26.894 |
| 6 | 489.1764 | 26.392 |
| 7 | 477.1762 | 23.401 |
| 8 | 525.2341 | 34.531 |
| 9 | 489.1762 | 23.373 |
| 10 | 508.2188 | 28.968 |
| 11 | 506.2028 | 29.121 |
| 12 | 646.3073 | 23.251 |


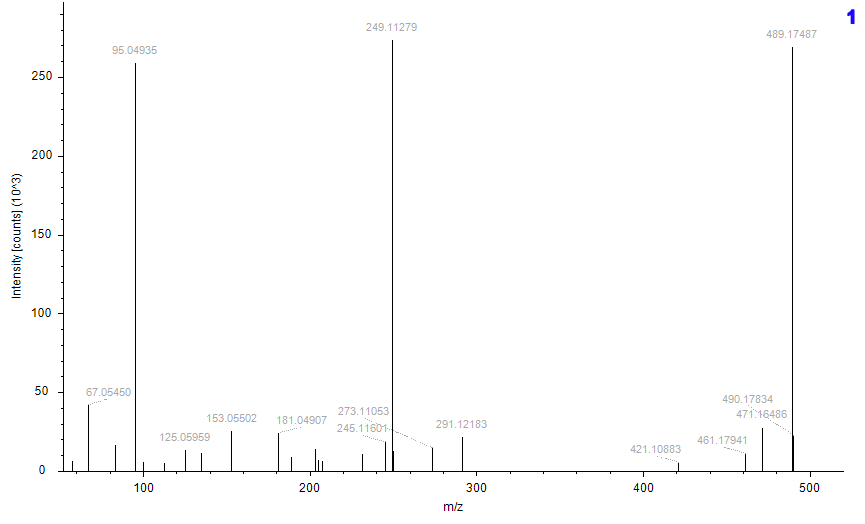

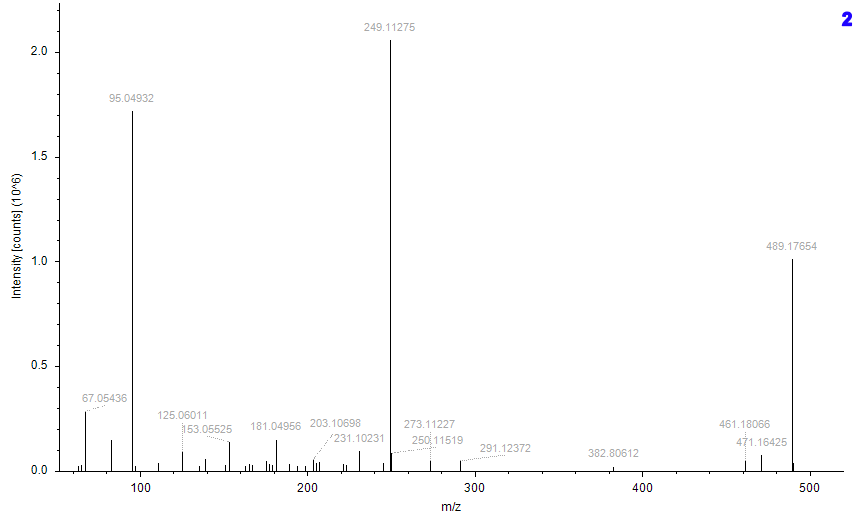


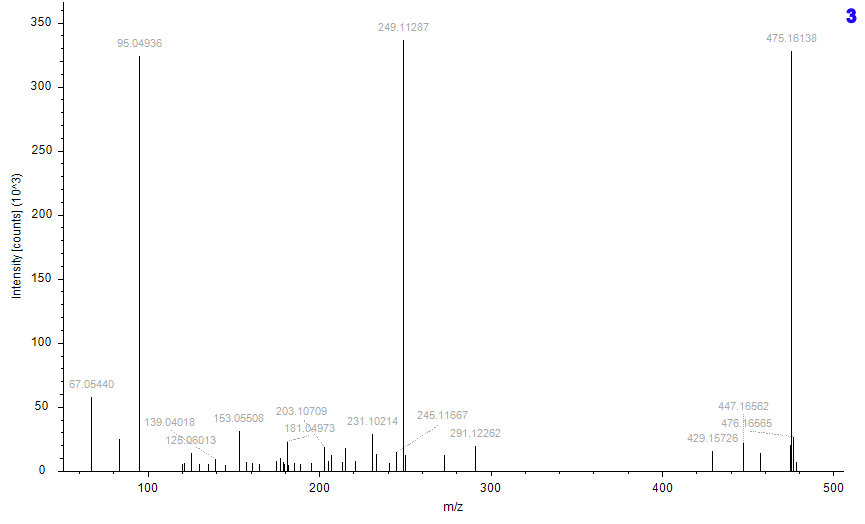

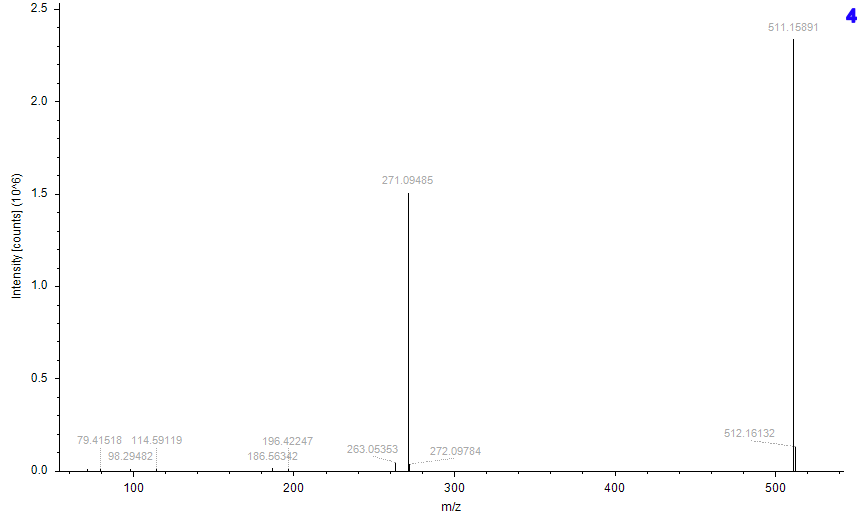


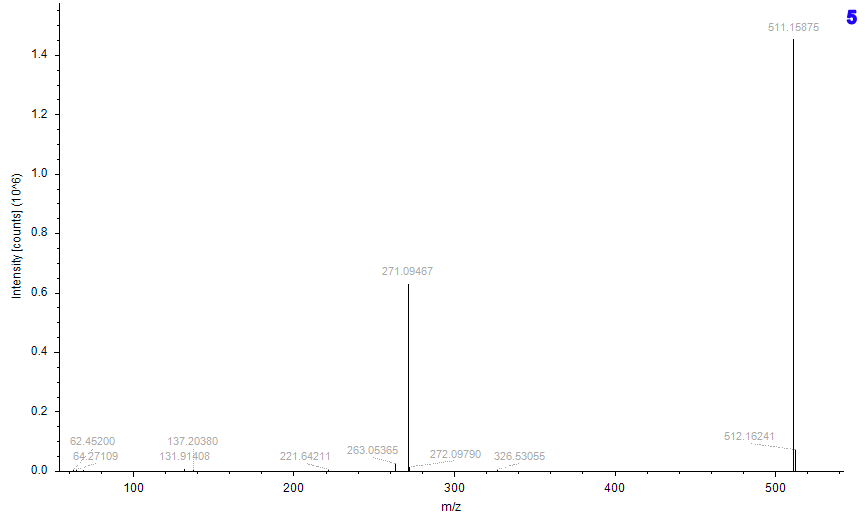

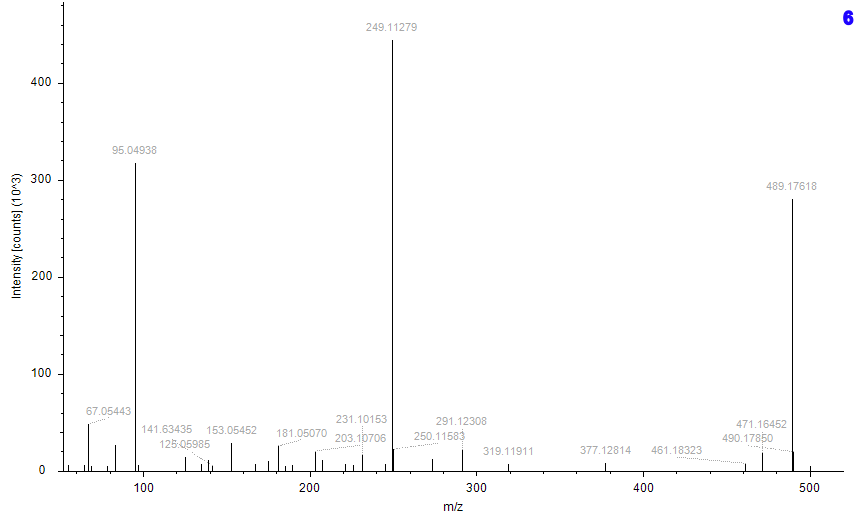

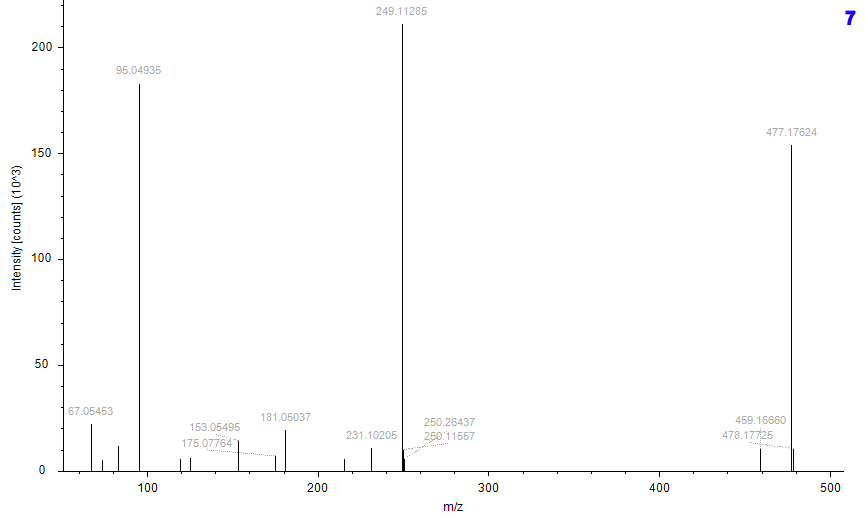

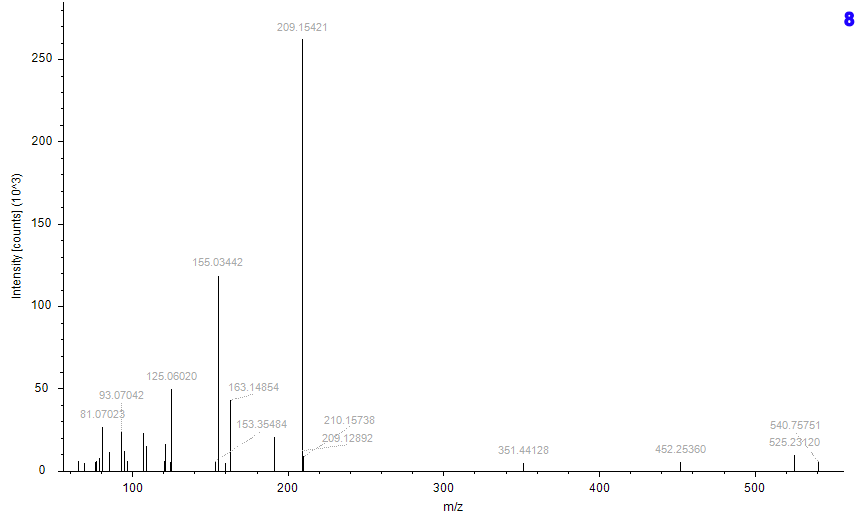


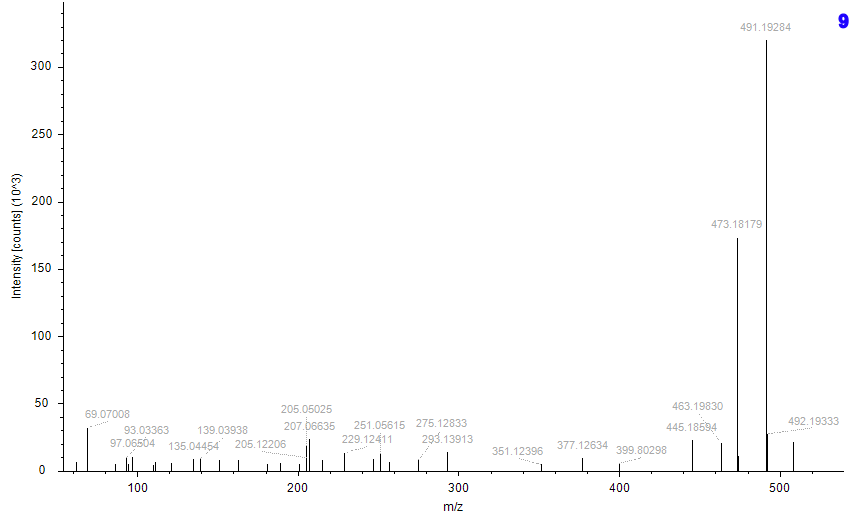

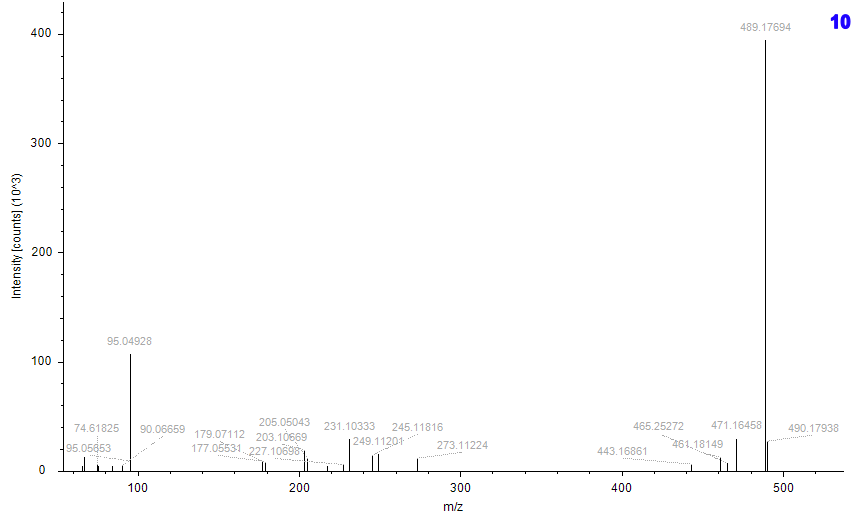

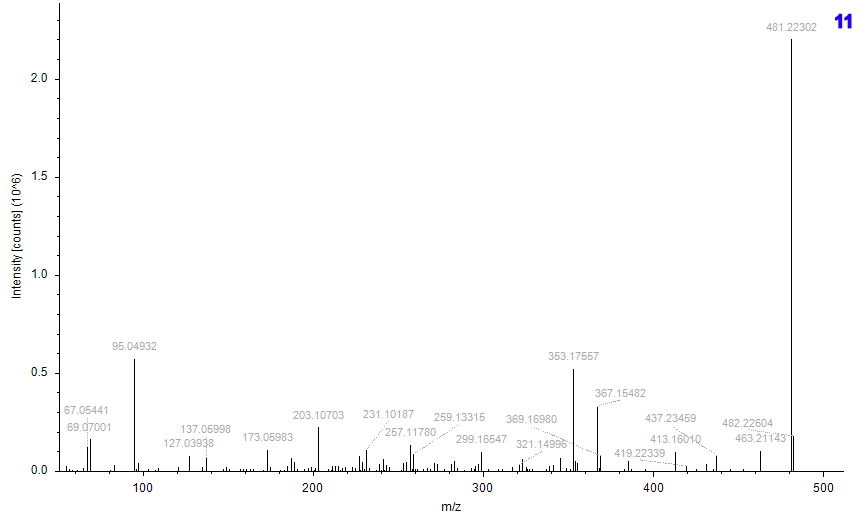

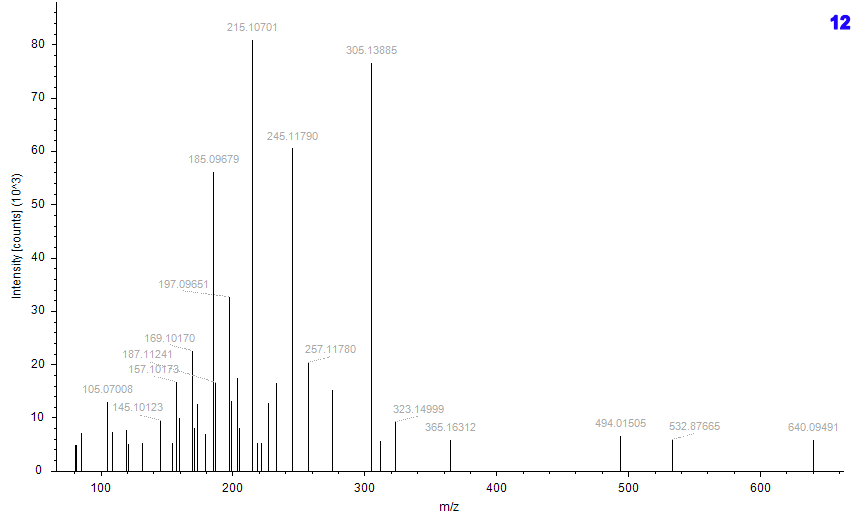


**Figure S2.** Fragmentation spectrum of the metabolites presented in Table S4.


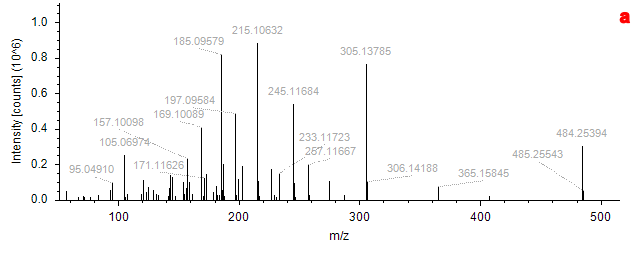


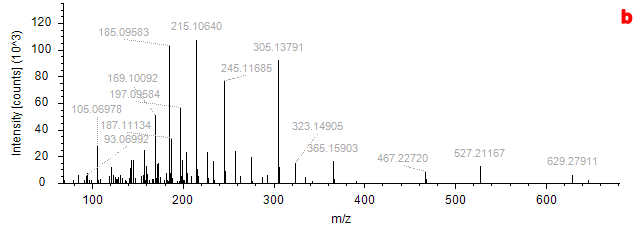


**Figure S3.** Fragmentation spectrum of (a) T-2 toxin, (b) T-2 toxin-α-glucoside.

**Table 4S.** Ions mass to charge ratio (*m/z*), retention time for individual mycotoxins, the correlation coefficient (R^2^), analytical ranges, limit of quantification (LOQ), and limit of detection (LOD).

| **Mycotoxin** | **Adduct** | **Precursor Ion** | **Rt [min]** | **R^2^** | **Analytical ranges [mg/kg]** | **LOQ [mg/kg]** | **LOD [mg/kg]** |
| --- | --- | --- | --- | --- | --- | --- | --- |
| T2 | [M+NH_4_]^+^ | 484.2529 | 27.92 | 0.9982 | 0.01-128.00 | 0.01 | 0.003 |
| T2-3α-G | [M+NH_4_]^+^ | 646.3060 | 24.26 | 0.9920 | 0.01-128.00 | 0.01 | 0.003 |
| T2-3β-G | [M+NH_4_]^+^ | 646.3060 | 24.50 | 0.9966 | 0.01-128.00 | 0.01 | 0.003 |
| HT2 | [M+NH_4_]^+^ | 442.2427 | 23.49 | 0.9470 | 0.02-64.00 | 0.03 | 0.01 |
| HT2-3α-G | [M+NH_4_]^+^ | 604.2969 | 21.28 | 0.9921 | 0.02-64.00 | 0.03 | 0.01 |
| HT2-3β-G | [M+NH_4_]^+^ | 604.2969 | 21.48 | 0.9954 | 0.02-64.00 | 0.03 | 0.01 |
